# Molecular Dynamics Workflows to Compute Large-Scale Sets of Absolute Binding Free Energies Aiding Drug Candidate and Binding Pose Selection

**DOI:** 10.1021/acs.jctc.5c02127

**Published:** 2026-05-13

**Authors:** Sebastian Wingbermühle, Akash Deep Biswas, Domenico Bonanni, Tatiana Shugaeva, Davide Gadioli, Jakub Beránek, Giorgia Frumenzio, Lara Querciagrossa, Andrea Piserchia, Gianmarco Accordi, Filippo Lunghini, Carmine Talarico, Andrew Emerson, Jan Martinovič, Andrea Rosario Beccari, Gianluca Palermo, Erik Lindahl

**Affiliations:** † Dept. Applied Physics, Science for Life Laboratory, 7655 KTH Royal Institute of Technology, Solna 17165, Sweden; ○ 18798 EXSCALATE, Dompé Farmaceutici S.p.A., Naples 80131, Italy; § Department of Physical and Chemical Sciences, 9303 Università degli Studi dell’Aquila, L’Aquila 67100, Italy; ∥ Data Science and Computation Facility, Istituto Italiano di Tecnologia, Genova 16163, Italy; ⊥ Dept. of Electronics, 18981 Information and Bioengineering, Politecnico di Milano, Milan 20133, Italy; # IT4Innovations, 563255 VSB − Technical University of Ostrava, Ostrava-Poruba 70800, Czech Republic; ∇ HPC Department, 18216 CINECA, Bologna 40033, Italy

## Abstract

Large-scale Virtual Screening (VS) campaigns of compound libraries can significantly speed up candidate selection in the early stages of drug discovery. The most promising drug candidates are identified by Scoring Functions (SFs), which enable VS campaigns to rank candidate compounds according to their estimated binding affinities. These SFs are typically trained on experimental data reflecting binding affinities (e.g., Dissociation Constant (*K*_d_) values), commonly used as proxies for protein–ligand binding free energies. Because experimental reference data are often unavailable or collected using inconsistent techniques and/or procedures between laboratories, we developed two computational workflows that generate configurational ensembles of soluble protein–ligand complexes with Molecular Dynamics (MD) and compute the Absolute Binding Free Energies (ABFEs) of the sampled ligand binding poses with implicit-solvent calculations. The resulting consistent large-scale datasets of ABFEs address two complementary aspects of virtual screening: quantitative binding affinity estimation and binding pose assessment. Our Binding Affinity Prediction (BAP) workflow estimated protein–ligand binding affinities for 4000+ complexes from the PDBbind 2020 dataset. Our Pose Selector (PS) workflow computed non-convergence ABFEs from short Molecular Dynamics (MD) simulations, estimating the stability of 800,000+ related binding poses. To produce ABFE data at this scale, our free-energy workflows classify, check, and repair input structures of protein–ligand complexes in a fully automated fashion. The workflow scripts, molecular dynamics data, and ABFE labels are publicly available, creating an extendable database of reference values for the development of Scoring Functions for Large-Scale Virtual Screening campaigns.

## Graphical abstract

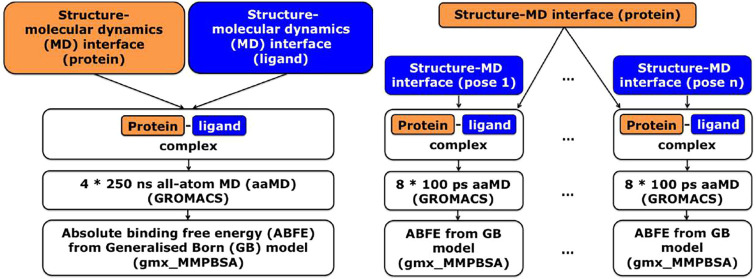

## 1 Introduction

The time from the identification of a new protein target to the market release of a new potent drug acting on that target is still usually measured in decades.^1^ To allow faster responses to emerging pathogens or diseases thus far without cure, computer-aided drug design aims at complementing human scientific intuition and time-consuming experimental screens with systematic virtual screens of compound libraries on large-scale computational resources.^2^ In the initial stages of the drug design process, the time needed to identify drug candidates can thus be reduced from years to months.^1^ In particular, scaffolds matching the geometry and chemistry of a target binding pocket and further optimisations of the resulting leads can be identified by systematically screening databases enumerating known, purchasable, synthesizable or theoretically possible small organic molecules against the binding pocket of interest in the target protein.^3–8^ If the databases link the screened drug candidates to available precursors from which they can be produced via known chemical reactions with high yields and selectivity, a reliable computational ranking of small organic molecules from exhaustive databases will enable the speedy verification of potent leads in the wet lab even if the drug candidate is not yet commercially available.^3,7^

Modern docking codes like LiGen,^9,10^ AutoDock-GPU,^11,12^ Uni-Dock,^13^ and DOCK 3.8,^14^ are already capable of screening billions or even trillions of drug candidates within hours if provided the necessary HPC or Cloud computational resources. However, to achieve this throughput, they have to rely on empirical, largely enthalpy-based scoring functions. These functions either ignore or model in a simplistic way entropic contributions to binding affinity, and they may fail to adequately account for solvation effects.^15^

So, their ability to identify the drug candidate with the highest binding affinity for the target protein (scoring/ranking power) as well as their ability to select the most stable binding pose of the resulting protein–ligand complex (docking power) could be further improved by using large-scale datasets linking structural ensembles of protein–ligand complexes to binding affinities. Experimentally determined binding affinities are an obvious source of reference data. However, experimental binding affinities may vary between laboratories and according to the protocol used, which can result in significant noise in the data.^16^ Moreover, experimental data are almost always ensemble averages that do not allow judging the stability of individual binding poses.^17^ To the best of our knowledge, no experimental counterpart can be used for training Machine Learning models for ligand Pose Selection. Established training labels typically exploit RMSD data, but these have been shown to provide Machine Learning models with limited accuracy.^18^ Within the limits of the accuracy of the force field employed and the amount of configurational sampling achieved,^19^ all-atom molecular dynamics simulations may provide a more consistent dataset of reference binding affinities than a collection of data from many different laboratories using many different protocols. Moreover, the sampling time in molecular dynamics simulations can be adjusted to control the number of ligand binding poses and protein configurations included in the binding affinity estimate, as described in detail below.

Ideally, datasets including many different and dissimilar protein–ligand complexes serve as reference datasets for the development of docking scoring functions, especially now that state-of-the-art docking scoring functions are frequently improved with the help of machine learning.^15,18,20^ To rank protein–ligand complexes in terms of binding affinity, relative binding free energies (RBFEs) of pairs of protein–ligand complexes could be used, which generally converge faster in MD simulations than ABFEs as long as the protein–ligand complexes compared are chemically similar. However, for the diverse datasets that we were interested in, there exists no set of pairs of chemically similar protein–ligand complexes that enables us to rank all protein–ligand complexes in the dataset. To cover the full dataset, a significant number of these pairs would have to contain highly dissimilar ligands or even highly dissimilar proteins. Consequently, the assumption that RBFE computations converge faster and yield more accurate results does not hold for our datasets because the alchemical perturbation in a significant number of pairs of protein–ligand complexes would be large, potentially too large to converge the respective relative free-energy calculation at all.^21^

Because they introduce a large chemical perturbation, it usually takes a significant amount of sampling time for an ABFE computation to converge, and ABFE estimates may suffer from systematic errors because contributions to the free energy are neglected.^22^ However, a docking scoring function does not aim to predict accurate ABFE estimates, but it has to provide an accurate ranking of ligands and binding poses. To achieve a good trade-off between precision and accuracy on the one hand and required computational resources on the other hand, we therefore decided to obtain configurational ensembles from all-atom molecular dynamics simulations to which ABFEs were assigned with implicit-solvent calculations.^23^ If not explicitly stated otherwise, throughout this work with “ABFEs” we refer to the above-mentioned estimates computed by means of implicit solvation models. We validated that the ABFE estimates yielded by our workflows provide the correct ranking of binding affinities of distinct protein–ligand complexes by studying their correlation with a test set of experimental binding affinities.^24^

In statistical thermodynamics, binding free energies are defined with the help of the partition function, i.e., an integral over the full configurational space, which can only be computed from simulations in the limit of infinite sampling.^25^ However, a distribution of configurations that is representative of the targeted Boltzmann distribution and allows to estimate binding free energies sufficiently accurate to support the drug design process can be obtained within finite sampling time.^22^ To compute ABFEs that serve as reference data to assess the scoring/ranking power of a docking scoring function (Figure 1), we therefore aimed to produce trajectories containing a diverse ensemble of ligand binding poses. In particular, the ensemble of configurations in the molecular dynamics trajectories must contain the relevant low-energy binding poses to provide a representative sample of the Boltzmann distribution of the respective protein–ligand complex in solution. For the protein–ligand complexes in our dataset, all-atom molecular dynamics simulations on the scale of hundreds of nanoseconds turned out to be necessary to sample distinct metastable ligand binding poses. During workflow validation, the exact simulated time per trajectory was chosen as the sampling time at which the correlation between the ABFEs computed with our workflow and the experimental reference values reached a plateau, indicating that additional configurational sampling no longer improved the quality of the ABFE estimate.

**Figure 1:**
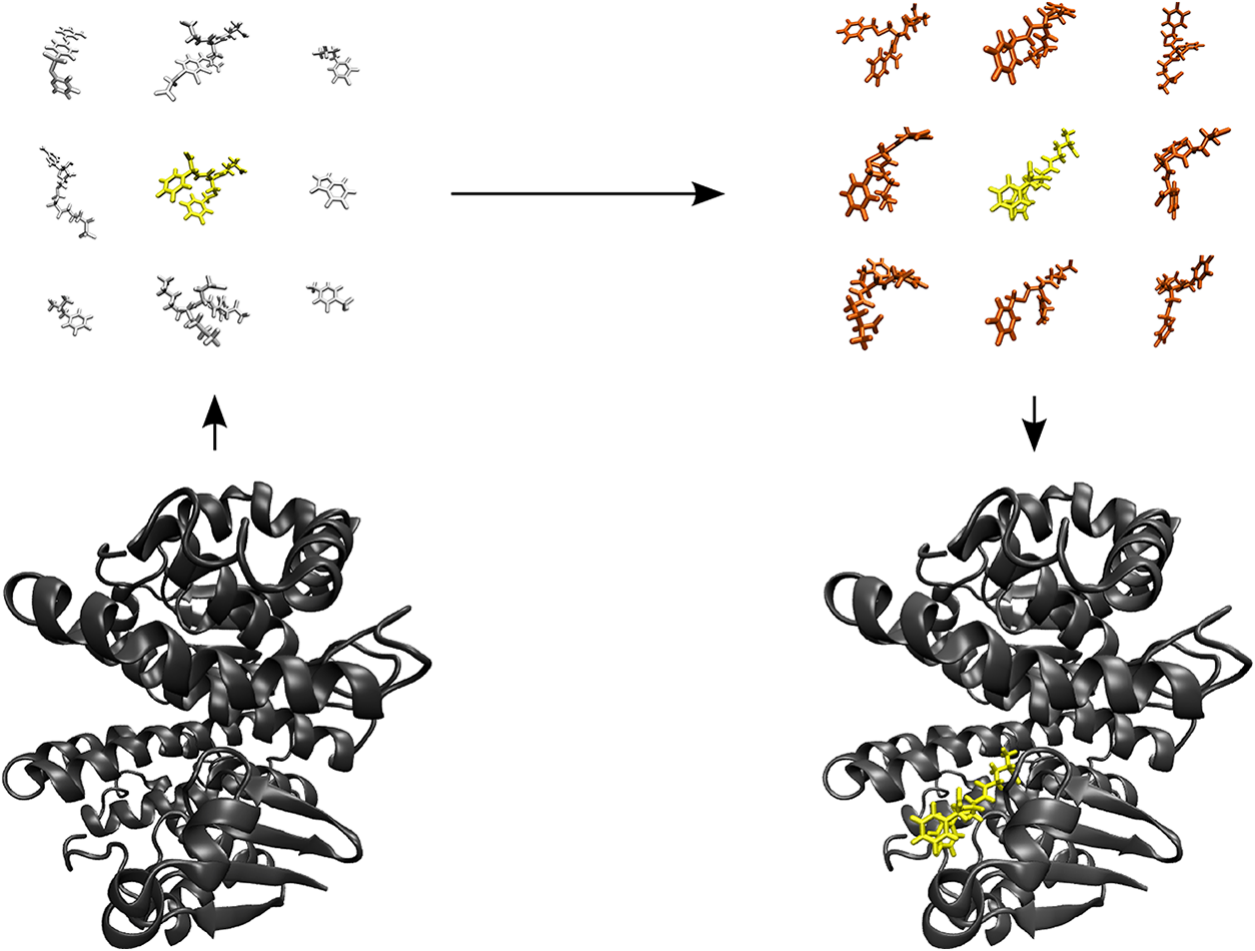
Virtual screening campaigns aim to computationally select the most stably binding ligand (gold) for a target protein (black) from a large set of drug candidates (light silver). However, molecular docking judges the affinity of a ligand on the basis of one or a few binding poses with the lowest scores (gold) while discarding less favourable binding poses (red-orange). To obtain reliable results with this approach, the scoring function must correctly capture binding affinity changes due to the chemical composition of the ligand (scoring/ranking power). Additionally, it must correctly rank different binding poses of the same ligand (docking power) such that both ligands are represented by their most favourable binding pose. Therefore, ligand selection can be split into two subproblems: identifying the ligand that, considering the ensemble of all possible binding poses and including entropic effects, has the lowest binding free energy (ranking power, top left) and finding the most (energetically) favourable binding pose of that ligand to obtain a realistic structure of the protein–ligand complex (docking power, top right).

In contrast, to assess the docking power of a docking scoring function, ABFE estimates that describe the stability of a single ligand binding pose are required. While a binding pose is technically one configuration of the ligand in the binding pocket, free energies are defined as integrals over phase space volumes to account for entropic effects, i.e., they, by definition, include more than a single configuration of the protein–ligand complex and the surrounding water molecules.^25^ Therefore, the underlying configurational ensembles should explore configurations that are different from, but still very similar to the initial binding pose. In particular, the configurational ensembles assigned to two binding poses under study must not overlap such that ABFE estimates can be assigned unambiguously. Therefore, we limited configurational sampling by using a very short simulated time of 100 ps to allow the system to only explore microstates lying in the immediate phase space neighborhood of the initial binding pose, i.e., only minor fluctuations of the initial binding pose were sampled.

Both ABFE estimates were determined with the help of implicit-solvent calculations. We selected implicit-solvent calculations because they can provide a good trade-off between accuracy, range of applicability and computational cost. Although further details are discussed in Section 2, a literature search suggested that implicit-solvent calculations were the most promising approach to produce a set of consistent and reliable ABFE estimates for the entire PDBbind 2020 refined set with the available computational resources. For example, the performance of Molecular Mechanics/Generalised Born Surface Area (MM/GBSA) and Molecular Mechanics/Poisson–Boltzmann Surface Area (MM/PBSA) implicit-solvation methods was assessed on more than 1800 protein–ligand crystal structures from the PDBbind database.^26^ It was demonstrated that the overall best Pearson correlation achieved was 0.579 ± 0.002 based on the minimized structures for the former and 0.491 ± 0.003 based on 1 ns molecular dynamics simulations for the latter.

Here, we present two workflows that take a protein structure and a ligand binding pose as input and return an ABFE estimate without requiring human scientific intervention. To achieve this, they provide solutions to four challenging problems: the automated preparation of input structures for molecular dynamics simulations, the assignment of force field parameters, the coordinated execution of many molecular dynamics trajectories and the cost-efficient computation of sufficiently accurate ABFEs. While good solutions addressing one of the problems exist, full workflows that provide an integrated solution to all four problems, that can be executed at this scale (millions of molecular dynamics simulations, hundreds of thousands of free-energy estimates) and that are fully public, including both workflow scripts and datasets produced with these workflows, are rare.

To prepare structures of protein–ligand complexes that can be used as input for molecular dynamics simulations, typical errors, such as structural gaps in the protein, must be found and fixed. Here, computational workflows can rely on established software suites used to check and process experimental protein structures or to predict protein structures with the help of homology modelling.^27–29^ However, there are very few tools that aim to prepare an experimental protein–ligand complex structure for molecular dynamics simulations with fully automated checks and error handling. Moreover, the protein and ligand need to be classified so that the correct molecular dynamics protocol is applied afterwards, e.g., membrane and soluble proteins require largely different simulation protocols, and input structures often need to be converted to be compatible with molecular dynamics force fields, e.g., due to naming conventions or non standard residues and ions that are not supported by the force field. For this task, the software tools with the highest technology readiness level currently belong to the proprietary Schrödinger software suite.

Regarding topology generation, standard molecular dynamics force fields like Amber come with software tools to assign force field parameters to biomolecules and small organic ligands,^30,31^ and wrappers to support the integration into workflows are available.^32,33^ In our workflows, we employ STaGE, a Python program that calls the force field-specific tools and wrappers and serves as an interface to obtain topologies for small organic molecules without using force field-specific command line syntax in the workflow scripts.

Large swarms of molecular dynamics trajectories are frequently employed to calculate kinetic quantities and explore reaction mechanisms with simulations. Consequently, solutions to efficiently manage swarms of trajectories on HPC clusters have evolved.^34–36^ However, the resulting software tools are designed to support specalised simulation protocols and cannot be easily applied to manage arbitrary swarms of trajectories. As a more general approach, runtime systems and meta-schedulers that coordinate the execution of large numbers of molecular dynamics simulations if they are expressed as jobs in a task graph, have been developed.^37,38^ Because molecular dynamics engines like GROMACS are highly optimised to use allocated computing resources efficiently, long trajectories can be managed in a resource-efficient manner with schedulers provided by the HPC centre like SLURM because the jobs producing the trajectories ideally occupy the same set of nodes without interruption up to the time limit of the HPC centre. However, sets of many short molecular dynamics simulations are executed most efficiently if a (meta-) scheduler is allocated the compute nodes and launches new simulations without returning the allocation to the scheduler used by the HPC centre. For this reason, we integrated the Pose Selector workflow with the metascheduler HyperQueue.^37^

Protocols computing binding free energies are a field of active method development,^39,40^ and the accuracy and precision of binding free energies obtained from molecular dynamics simulations are tested regularly, e.g., in the SAMPL challenges.^41^ However, if the free-energy protocol is based on all-atom molecular dynamics, development efforts often focus on computing accurate free energies for few complex systems that pose sampling challenges like very slow motions or require the protocol to adapt key simulation parameters without human intervention.^21,42,43^ The development of efficient free-energy protocols and workflows for high-throughput screens, in contrast, is still at an early stage.^39^ Nevertheless, several studies in which protein–ligand binding free energies were calculated at large-scale on both HPC clusters and cloud resources have recently been published, obtaining decent correlation with experimental values and competitive accuracy compared to proprietary software packages.^44–47^ In these studies, binding free energies were estimated for hundreds of protein–ligand complexes. To obtain absolute binding free energies for thousands of complexes, so far almost only implicit-solvent methods have proven affordable, although with modelling constraints that reduced the computational requirements but also the expected accuracy.^26,48^ For example, the performance of Molecular Mechanics/Generalised Born Surface Area (MM/GBSA) and Molecular Mechanics/Poisson–Boltzmann Surface Area (MM/PBSA) methods has been assessed by Sun et al. on more than 1800 protein–ligand crystal structures from the PDBbind database.^26^ Finally, in an exceptional effort to discover a drug against COVID-19, ABFEs were also calculated using free-energy perturbation, i.e., all-atom molecular dynamics, for approximately 12,000 protein–ligand complexes.^49^

Few open-source tools are available to compute both absolute and relative binding free energies in an automated fashion. The pmx software suite allows to automate the generation of topologies and the subsequent free-energy computation using non equilibrium thermodynamic integration, which was successfully tested at the scale of hundreds of protein–ligand complexes.^44–47,50^ Other interesting examples are the automated workflow for production free-energy simulation setup and analysis (ProFESSA) relying on the GPU-accelerated AMBER free-energy engine to effectively calculate absolute and relative solvation free energies and relative binding free energies,^51^ and BAT.py, a Python tool making use of the AMBER simulation package to automate the calculation of binding free energies for a protein with a series of ligands.^52^ However, although such methods minimize human efforts and still provide satisfying results, they are still unsuited to calculate absolute binding free energies for thousands of dissimilar complexes. For example, the pmx software suite, while a powerful and established tool for alchemical RBFE calculations, faces significant limitations when applied to calculating ABFEs for thousands of structurally dissimilar complexes, primary challenges lying in the computational expense of ABFE, structural dissimilarities, sampling requirements, and setup complexity. Similarly, BAT.py is unsuitable for calculating ABFEs for thousands of dissimilar complexes due to a combination of high computational costs, convergence issues for diverse chemistry, and technical limitations in handling large-scale, automated docking pose optimisation. In fact, while BAT.py automates the workflow for ABFE calculation, it can primarily serve as a “high-cost” re scoring tool, but fundamentally inefficient for high-throughput screening of massive, diverse datasets. Finally, while ProFESSA enhances the automation and sampling of alchemical free energies (e.g., using ACES) in RBFE calculations, calculating ABFEs for a diverse set of complexes using this approach is fundamentally more demanding than it is for calculating RBFEs for a series of congeneric ligands. For thousands of dissimilar complexes, the cost of fully decoupling each ligand in complex and solvent becomes prohibitive, since this process would require many independent simulation windows to ensure the accuracy of the coupling/decoupling steps, at the risk of poor convergence. Last but not least, while GPUs have made calculations faster, dissimilar compounds often have different binding modes or require significant conformational changes to bind, which are difficult to be properly sampled during an automated ABFE process.

In this paper, after summarising the theory of implicit-solvation models and explaining our main design decisions for the implicit-solvent calculations in our workflows (Section 2), we describe the goals and architecture of the resulting Binding Affinity Prediction (BAP) and Pose Selector (PS) workflows. Next, we present in detail the structure-molecular dynamics interface that prepares the protein–ligand complexes provided as workflow input for the molecular dynamics simulations (Section 4.2), before discussing the validation and large-scale execution of the molecular dynamics simulations (Section 4.3). Then, the validation of our protocol for the implicit-solvent calculations is described in detail (Section 4.4), followed by the results of the large-scale execution on the PDBbind 2020 refined set (Section 4.5) and of initial machine learning tests using the binding free energies computed with our workflows as training data (Section 4.6). In the conclusions (Section 5), we briefly describe the fully public datasets produced with the BAP and PS workflows and summarise their potential benefits for the development and improvement of both docking scoring functions and molecular dynamics-based free-energy methods.

If you read the Results and Discussion (Section 4) in chronological order, you follow the key tasks performed by the workflows (generation of simulation input files, molecular dynamics simulations, implicit-solvent calculations), and for each task, our validation efforts are discussed before the results of the large-scale execution are presented.

## 2 Theory

Available computational methods for calculating binding affinities for small molecules in protein targets range from fast docking techniques to highly accurate (but computationally expensive) free energy simulations. At the time of writing, methods widely used in drug discovery to calculate (relative and absolute) binding affinities include:

- Molecular Docking, which makes use of empirical, physics-based or knowledge-based scoring functions to estimate the expected orientation of a ligand within the binding site, together with its binding affinity. It is the least computationally demanding technique and, as such, it is largely exploited in the first stages of High Throughput Virtual Screening (HTVS) campaigns.
- End-point Free Energy Methods, like MM/PBSA and MM/GBSA), which calculate binding free energies of a ligand-protein complex by relying on its energy-minimized structure or averaging non bonded interactions over snapshots of a molecular dynamics trajectory. They are termed “end-point” because they calculate the binding free energy by evaluating the ligand, receptor, and complex only at their end points, as opposed to path-sampling methods. In addition, by using implicit solvation models they offer a balance computational efficiency and accuracy, often outperforming docking scoring functions in the latter.
- Path-sampling methods like Free Energy Perturbation (FEP) and Thermodynamic Integration (TI) are physics-based techniques for computing free energy differences by creating unphysical, intermediate states connecting initial and final states by means of 8a collective variable, usually called λ. Calculating the free-energy difference between the two end states usually involves introducing multiple intermediate states (with more manageable free-energy differences),^53^ which can be sampled in ergodic systems through Molecular Dynamics (MD) or Monte Carlo simulations.^54^
- Quantum Mechanics/Molecular Mechanics (QM/MM) methods, which employ QM for the binding site and MM for the rest of the system to achieve high-accuracy predictions, particularly when electronic structure changes are significant.
- Hybrid Neural Network Potentials/Molecular Mechanics (NNP/MM) methods, an evolution of the previous class combining the high accuracy of NNPs, offering near-quantum chemical (QM) accuracy for intramolecular forces, with the computational efficiency of Molecular Mechanics (MM) methods, for treating the surrounding solvent and protein environment.

Among established computational methods to obtain absolute binding free energies (ABFEs) for protein–ligand complexes, implicit-solvation models offer a good compromise between throughput and accuracy when thousands of complexes have to be dealt with.^26,48^ End point methods like MM/GBSA, MM/PBSA and MM/3D-RISM require way less constraints to be obeyed than more theoretically rigorous (and computationally expensive) alchemical free-energy estimation methods (also called pathway methods).

On the other hand, less computationally intensive implicit-solvation models are used to predict ABFEs from snapshots extracted from all-atom MD trajectories modelling water molecules explicitly. The explicit waters are removed, the solute is placed in a dielectric continuum, and the electrostatic component of the interaction between the solute and the solvent is calculated. By adding non polar contributions, usually empirical terms accounting for the energy needed to create a cavity for the solute and for van der Waals interactions, the free energy of solvation is obtained.

Finally, the binding free energy is calculated as

$$\left\langle \Delta G_{\mathrm{bind}}\right\rangle =\left\langle \Delta G_{\mathrm{RL}}\right\rangle -\left\langle \Delta G_{R}\right\rangle -\left\langle \Delta G_{L}\right\rangle$$

where the subscripts R, L, RL denote receptor, ligand and complex quantities. If each free energy term on the right-hand side of the equation is obtained from an individual simulation, the multiple-trajectory protocol (MTP) is used, where separate simulations of the unbound protein, the unbound ligand and the protein–ligand complex are performed. However, in practice, it is far more common to generate trajectories for the protein–ligand complex only and then obtain the three free energies at hand from this single simulation. With this approach, called single-trajectory protocol (STP), the precision of the method is improved, and the intramolecular energy terms *E*_int_ (e.g., bonded energy terms) cancel out. However, the STP approach ignores structural changes of the ligand and the receptor upon binding and may thus be unsuitable for systems that undergo induced fit. In contrast, the MTP approach more accurately addresses the role of the adaptation energy, i.e., the free energy associated with conformational changes upon induced fit binding.^55,56^ On the other hand, depending on the tested system, the MTP approach can bring large uncertainties, up to several kcal/mol.^57^ In fact, when dealing with a large number of chemically diverse complexes, the STP approach often gives more accurate results than the MTP approach when the protein, ligand, protein–ligand states are not dissimilar, i.e., in the absence of induced fit, when binding is mostly mediated by a lock-and-key mechanism.^58^

In the context of MM/PBSA or MM/GBSA, the binding free energy Δ*G*_bind_ is calculated according to the following equations:

$$\{\begin{array}{l} \Delta G_{bind}=G_{RL}-\left(G_{R}+G_{L}\right)=\Delta H-T\Delta S=\Delta E_{MM}+\Delta G_{sol}-T\Delta S\\ \Delta E_{MM}=\Delta E_{internal}+\Delta E_{electrostatic}+\Delta E_{vdW}\\ \Delta G_{sol}=\Delta G_{p}+\Delta G_{np}\\ \Delta G_{np}=a\times \mathrm{SASA}+b \end{array}$$

where the binding free energy Δ*G*_bind_ has been decomposed into three terms:

- the molecular mechanics energy (Δ*E*_MM_), which is used to compute the energy of the direct interaction between the protein and the ligand and is the summation of the:
  - intramolecular energy (Δ*E*_internal_), including bond, angle, and dihedral energies,
  - electrostatic energy (Δ*E*_electrostatic_),
  - van der Waals energy (Δ*E*_vdW_).
- the solvation free energy (Δ*G*_sol_), which is obtained from the implicit-solvation model and made up of:
  - a polar (Δ*G*_*p*_) contribution, estimated by means of either the Generalised-Born or Poisson–Boltzmann model,
  - a non polar (Δ*G*_*np*_) contribution that is usually assumed to be proportional to the molecule's total solvent accessible surface area (SASA), with a proportionality constant derived from experimental solvation free energies of small non polar molecules.^59^
- an entropic contribution (*T*Δ*S*) that takes into account the configurational entropy change upon binding. It is added to the solvation free energy as a correction after applying the implicit-solvation model.

So we finally get:

$$\Delta G_{bind}=\Delta E_{internal}+\Delta E_{electrostatic}+\Delta E_{vdW}+\Delta G_{p\left(\mathrm{GB}/\mathrm{PB}\right)}+\Delta G_{\mathrm{np}\left(SASA\right)}-T\Delta S$$

One reason for applying an implicit-solvation model to calculate the free energy of a system is to avoid the large fluctuations of the potential energy that are caused by the explicit water molecules present in the trajectory. Consequently, the solvation models and, thus, the Hamiltonians, are different in the simulation and in the post-processing phases.^60^ Therefore, every configuration in the trajectory should, strictly speaking, be reweighted with the Boltzmann factor of the difference between the energies with the post-processing Hamiltonian (implicit solvent) and the simulation Hamiltonian (explicit solvent). However, both Hamiltonians agree on the interaction energy between the protein and the ligand such that the composition of the configurational ensemble of the protein–ligand complex is not expected to change significantly if the weights of the individual configurations are updated from the explicit-solvent Hamiltonian to the implicit-solvent Hamiltonian. For this reason, the configurational ensemble obtained in the all-atom MD simulation is usually not reweighted in practice.

The generalised Born (GB) and Poisson–Boltzmann (PB) models differ in the way that Δ*G*_*p*_ is computed. The MM/PBSA method attempts to calculate *E*_*p*_ by numerically solving the PB equation, which describes the electrostatic interactions that a solute undergoes when it is placed in a solvent containing ions:

$$\mathrm{\nabla ⃗}\cdot \left[\epsilon \left(\mathrm{r⃗}\right)\mathrm{\nabla ⃗}\Psi \left(\mathrm{r⃗}\right)\right]=-\rho \left(\mathrm{r⃗}\right)-\sum_{i=1}^{N}c_{i}^{\infty }q_{i}q\lambda \left(\mathrm{r⃗}\right)e^{-z_{i}q\Psi \left(\mathrm{r⃗}\right)/k_{B}T}$$

The PB equation is a three-dimensional, second order, non linear, elliptic, partial differential equation for which various methods to model the solute–solvent dielectric interface are available, including the geometric approach,^61^ level set functions^62^ and the immersed interface method.^63^ Still, the full PB equation is quite computationally expensive to solve. So, when both the ionic strength and the electric field are weak,^64^ its linearized form, in which the exponential is replaced with its first-order Taylor expansion, is usually introduced and solved.

The generalised Born (GB) implicit-solvation model is a fast but approximate technique to calculate molecular electrostatics in a solvent as described by the (linearized) Poisson–Boltzmann equation, which models water as a dielectric continuum. In the GB model, atoms are described as charged spheres whose internal dielectric constant is lower than that of the external solvent. The more an atom is surrounded by other atoms, the less its electrostatics will be screened since it is surrounded by lower dielectric material than, e.g., water. This property is called “descreening”, and GB models are based on evaluating atomic descreening.

More in detail, the GB model describes the solute as a set of spheres in a surrounding solvent characterized by a continuum dielectric constant ϵ_*w*_. According to this model, the solvation free energy can be written as

$$G_{solv}=-\frac{1}{2}\left(1-\frac{1}{\epsilon_{w}}\right)\sum_{i,j}^{N}\frac{q_{i}q_{j}}{r_{GB}}$$

where:

- ϵ_*w*_ is the absolute dielectric constant of the solvent;
- *r*_*i*,*j*_ is the distance between atoms *i* and *j*;
- *q*_*i*_, *q*_*j*_ are the partial charges of atoms *i* and *j*;
-
$r_{\mathrm{GB}}=\sqrt{r_{\mathrm{ij}}^{2}+a_{i}a_{j}\exp\left(-\frac{r_{\mathrm{ij}}^{2}}{4a_{i}a_{j}}\right)}$ , where *a*_*i*_, *a*_*j*_ are the Born radii of charged atoms *i*, *j*, respectively, and represent each atom's degree of burial within the solute.^65^ The effective radius of an atom is defined as the radius of a corresponding spherical ion having the same Δ*G*_*p*_ as the self-energy of this atom in the molecule. Here, the self-energy is defined as the polar solvation free energy of the molecule with partial charges set to zero for all atoms except the atom of interest. It is worth noting that the effective radius of an atom is larger than the intrinsic radius of its atomic sphere because of the descreening effect of surrounding atoms, reducing the extent to which the atom charge is screened by solvent.

We finally evaluated an attractive approach based on statistical mechanics and liquid state integral equation theory (IET).^66,67^ It starts by assuming explicit solvation, but it operates with 3D distributions of solvent molecules in the statistical ensemble rather than with molecular trajectories to predict the structure and thermodynamics of biomolecules in solution. It does so by solving the Ornstein–Zernike equation to obtain pair-correlation functions, from which different thermodynamic properties can be calculated. More in detail, we tested a particular version of the integral equation theory that calculates 3D solute–solvent correlation functions and is named 3D-reference interaction site model (3D-RISM). To solve the Ornstein–Zernike equation, we used the Kovalenko-Hirata (KH) closure relation as provided by gmx MMPBSA. The 3D-RISM method is eventually combined with molecular mechanics (MM) to generate the MM/3D-RISM method.^68^

## 3 Methods

Absolute binding free energy (ABFE) predictions by implicit-solvation models are sensitive to the value of the solute dielectric constant.^69,70^ Typical values to account for diversely charged pocket environments are^23^

- 4, for highly charged binding interfaces,
- 2, for two or three charged residues in the binding pocket and
- 1, for hydrophobic pockets.

MM/PBSA's performance is more sensitive to the investigated system than MM/GBSA's.^69^ While the MM/PBSA model can be considered more theoretically rigorous than MM/GBSA, ABFE estimates by MM/PBSA were reported to be highly sensitive to the value of the solute dielectric constant used for the investigated system.^71^ In contrast, MM/GBSA was found to be more robust and accurate when applied to a chemically diverse set of complexes like the PDBbind 2020 refined set. For example, a study assessed the performance of the MM/PBSA and MM/GBSA models in predicting ABFEs for more than 1800 (metal-free) crystal structures from the PDBbind set and concluded that, for very large and diverse sets of systems, MM/GBSA performs better than MM/PBSA.^26^ MM/GBSA can be used in multi-target comparisons, whereas MM/PBSA is more suitable for individual-target-level binding free energy rankings. MM/GBSA is therefore the preferred implicit-solvation model for an automated workflow that aims to obtain reliable ABFE estimates for all complexes in the PDBbind 2020 refined set without the system-specific optimisation of parameters like the dielectric constant.

Furthermore, the linearized PB equation is not suitable for highly charged systems,^64^ and both linear MM/PBSA and MM/GBSA were reported to provide inaccurate ABFE estimates for binding pockets accepting ligands with high formal charges (i.e., >1).^26^ However, it was shown that a solute dielectric constant higher than the one used in molecular dynamics (i.e., 4 instead of 1) often leads to better agreement with experiment.^72^ This is because the introduction of a higher solute dielectric constant is a reasonable but rough way to account for the screening of electrostatic interactions due to polarization of electronic, geometrical and solvent-exchange origins. When the dielectric constant is increased to 20, the performance measures remain quite similar to those of calculations with a dielectric constant of 4, with clear improvement only for highly charged binding pockets. These findings suggest that a higher solute dielectric constant may render MM/PBSA and MM/GBSA less sensitive to the formal charge of the binding pocket and the ligand in the investigated system.

To adapt to the environment of the binding pocket, variable dielectric models are a promising solution because they adjust the value of the internal dielectric constant. For example, VSGB 2.0 ^73^ approximates the solvation free energy with an optimised implicit-solvation model, which is based on the surface generalised Born approach (SGB) and the variable dielectric (VD) treatment of polarization from protein side chains. Because we handled thousands of protein–ligand complexes in this work and aimed at keeping computational costs and parametrisation efforts minimal, we benchmarked models using a single dielectric constant value.

Even with a higher solute dielectric constant that is usually chosen to account for highly charged environments in the binding pocket, coordinated metal ions can significantly impact the accuracy of ABFE estimates by MM/GBSA and MM/PBSA. The accuracy of implicit-solvation models depends on parameters like atomic radii and dielectric constants, which may not be well-defined or transferable for metal ions with varying coordination environments.^74,75^ With these parameters, the models cannot fully capture the directional and specific interactions between metal ions and coordinated protein residues, ligands and water molecules that are crucial for the stability of the complex. In particular, the MM/GBSA model simplifies the calculation of the electrostatic solvation free energy by approximating the Born radius of an atom. However, this approximation can be poor for ions, especially when they are highly charged or have a small ionic radius.^70,76^ As discussed in the context of the solute dielectric constant, the accuracy of MM/PBSA is again highly affected by the choice of parameters like the dielectric boundary and the treatment of the ionic strength. These limitations can lead to inaccurate predictions of binding affinities for metal-containing complexes such that these complexes are often excluded from the dataset.^26,74,75^

To efficiently use our computational resources, and since we were mostly interested in finding active ligands (ranking) rather than accurately reproducing experimental binding affinities (scoring), we decided to not include configurational entropy contributions in our binding free energy estimates. Actually, it was shown that the inclusion of the configurational entropies predicted by the truncated normal-mode analysis actually compromises the overall accuracy of MM/GBSA and MM/PBSA evaluated on a test set of more than 1500 protein–ligand systems.^48^ In another study, too, MM/GBSA and MM/PBSA could still achieve satisfactory accuracy of ranking without considering configurational entropy,^69^ and the increase in accuracy rarely justifies (e.g., in case of induced fit) the corresponding increase in computational cost to obtain converged estimates of the configurational entropy. Furthermore, other commonly used estimators have known shortcomings that would prevent them from providing reliable entropy estimates for the protein–ligand complexes of the PDBbind 2020 refined set. The quasi-harmonic approximation can only calculate an upper limit instead of an exact estimate of the configurational entropy.^77^ The interaction entropy approach relies on an exponential average that cannot be converged given the fluctuations of the interaction energy that are to be expected for the protein–ligand complexes under study, while C2 entropy estimates are expected to be significantly too high under these circumstances.^78^

We also benchmarked the effect of the length of the MD simulation on the quality of the binding free energy estimates. Minimized structures often give ABFE estimates as good as or even better than those obtained with (very) short MD simulations^79,80^ although some studies have emphasized the importance of MD sampling.^70^ Of course, predicting binding free energies from minimized structures will save much computational effort, but it would also ignore dynamical effects, making the results strongly dependent on the starting structure, and all information about the statistical precision of the method would be lost. On the other hand, increasing the simulation time beyond few tens of nanoseconds did not always improve the correlation between the predicted binding free energies and the experimental values,^60,81^ which sometimes is also due to force field inaccuracies. The influence of the force field and configurational sampling on the prediction performances of both MM/GBSA and MM/PBSA was also assessed for a test set of 46 small molecules targeting five different proteins.^81^ It was shown that the force field that is optimal for a given implicit-solvation model changes with the length of the MD simulation. As a trend, MD simulations longer than 1 ns are helpful to achieve reliable predictions with the Amber force fields ff99, ff99SB, ff99SB-ILDN, and ff12SB, but MD simulations of 1 ns or shorter are preferred for the ff03 force field. However, several combinations of implicit-solvation model, force field and trajectory length resulted in comparable performance such that no protocol could be identified that is expected to generally yield more accurate ABFE estimates. In addition, studies in the literature that judged the effect of the MD force field and configurational sampling did not extend the trajectories to several hundreds of nanoseconds, which are needed to sample slow motions of the ligand and surrounding protein residues in the binding pocket or larger conformational changes of the protein that may affect the binding pocket allosterically. This time scale was explored in our work.

For the Binding Affinity Prediction (BAP) workflow (see Section 4.1), 5316 experimental protein–ligand complex structures, as provided in the PDBbind 2020 refined set, were used as the workflow's input. For the Pose Selector (PS) workflow (see Section 4.1), up to 256 docking poses were generated using LiGen for each complex in the full PDBbind 2020 dataset and added to the experimental ligand binding pose of the complex.^18^ From this dataset, we used the binding poses of 5769 complexes as input for the PS workflow, extending the refined set with some complexes from the full PDBbind 2020 dataset.

To generate the additional binding poses for the PS workflow, the ligand was redocked inside its binding pocket, using the coordinates of the centroid of the crystal structure to identify the binding site. Each generated pose was characterized by its RMSD with respect to the ligand binding pose in the crystal structure.

In principle, the PS workflow can be applied to docking structures of protein–ligand complexes generated by any docking tool. LiGen was chosen as the docking engine for two main reasons. First, it is well suited to extreme-scale docking simulation,^9^ and second, docking poses can be generated by using purely geometric scoring, therefore excluding strong bias from the scoring function. This latter point was not possible with other tools, such as Glide, Plants, Gold or AutoDock. Excluding the scoring function was important to provide the PS workflow with a representative number of wrong samples, i.e., poses with an RMSD > 3 Å. More information related to the generated dataset can be found in a previous publication.^18^

These complexes and binding poses were classified, checked, repaired if necessary, and assigned AMBER force field parameters by the structure-MD interface. In the BAP workflow, four replicas of 250 ns MD simulations with GROMACS 2023.2 were run for each complex; in the PS workflow, eight replicas of 100 ps MD simulations were produced for each pose. In the subsequent implicit-solvent calculation with gmx_MMPBSA 1.6.1, the generalised Born (GB) model was used to estimate an absolute binding free energy (ABFE) for the complex or binding pose. In the BAP workflow, the ABFE assigned to each trajectory was averaged over 1251 equally spaced snapshots extracted every 200 ps; in the PS workflow, the ABFE of the final time frame of the trajectory was used. The final free-energy estimate for the complex or binding pose was obtained by averaging the ABFEs assigned to the individual trajectories over the replicas and using the standard error of the mean to estimate the statistical error.

The Pose Selector (PS) workflow was implemented and executed using HyperQueue,^37^ an HPC-optimised task runtime meta-scheduler designed for efficient execution of task graphs on supercomputers. It allows defining and submitting task graphs in an easy way, without forcing users to split their task graphs manually into separate Slurm allocations. The PS MD simulations were submitted as separate HyperQueue tasks that were scheduled to separate computational nodes on the LUMI supercomputer; GROMACS then took care of managing all the allocated GPU resources on individual nodes.

Two HyperQueue features were crucial for the PS workflow. The first was HyperQueue's metascheduling mechanism. HyperQueue performs fully dynamic metascheduling of tasks on top of Slurm (or PBS) allocations; it is able to automatically load balance tasks across separate allocations in response to current computational load. It also provides comprehensive support for resource management; tasks can specify arbitrary resource requirements, which are respected by the HyperQueue scheduler when assigning tasks to computational nodes. Thanks to the flexible and automatic mapping of tasks to allocations, it can execute a large amount of tasks in a small number of allocations, which allows it to overcome (frequently very strictly configured) allocation submission limits of Slurm. This allowed us to express the computed task graph in a natural way, without having to consider how it will be mapped to separate Slurm allocations. We did not even have to submit allocations manually because HyperQueue can automatically submit allocations on behalf of the user based on current computational demands.

The second feature of HyperQueue that was useful for this workflow was its built-in fault tolerance. The HyperQueue server, which keeps the state of submitted task graphs, was deployed as a persistent process running on a login node of the LUMI supercomputer, rather than running in ephemeral allocations. It was thus able to automatically reschedule tasks that have failed to execute (e.g., because of a Slurm allocation expiring in the middle of the computation), without the user having to intervene. This was crucial because of the large amount of submitted tasks and the relatively high failure rate caused by the instability of the LUMI cluster.

Without HyperQueue, it would have been quite challenging to submit such a large amount of tasks (about 6.5 M simulations in total) without having to manually handle resubmission of failed tasks and assignment of tasks to different allocations. Using HyperQueue also made the workflow portable; the only thing that needs to change (from the point of view of HyperQueue) to execute the workflow on a different supercomputing cluster are the credentials required for submitting Slurm or PBS allocations.

Further details are provided in Section 1 of the Supporting Information.

## 4 Results and Discussion

### 4.1 The Architecture of the Workflows

With the considerations detailed in the Introduction (Section 1) in mind, we designed the Binding Affinity Prediction (BAP) workflow to estimate ABFEs that allow assessing the scoring/ranking power of a docking scoring function and the Pose Selector (PS) workflow to produce ABFE estimates that allow assessing its docking power (Figure 2). The two workflows only support soluble proteins without membrane-spanning regions as input. In both workflows, the structure-molecular dynamics (MD) interface assigns force field parameters to the protein–ligand complex, solvates it in explicit water and adds ions to the system to provide a topology and starting structure for the subsequent MD simulations. However, the interface only completes these steps if the protein–ligand complex is classified as within the workflow’s scope, passes extensive quality checks, and errors in the structure can be repaired if necessary. For the fully prepared protein–ligand complex, all-atom MD simulations with explicit solvent are performed using GROMACS (version 2023.2). The BAP workflow aims to produce a well-sampled, representative configurational ensemble of all relevant ligand binding poses of the given protein–ligand complex in four replicas of 250 ns MD simulations. On the basis of this configurational ensemble, the BAP workflow later assigns one ABFE to the protein–ligand complex, estimating the binding affinity of the ligand to the binding pocket on the protein. In contrast, the PS workflow contains a loop over all available ligand binding poses and only tries to sample the immediate phase space neighborhood of the input binding pose in a statistically precise manner with the help of eight replicas of 100 ps MD simulations. Using these simulations, the PS workflow later computes the ABFE of each ligand binding pose individually, estimating the stability of the respective binding pose. In both workflows, the protein–ligand complexes are simulated in explicit water to capture the effect of surrounding water molecules and coordinated ions on ligand binding accurately in the configurational ensembles that are analysed in the implicit-solvent calculations. After being extracted from explicit water, the protein–ligand complex is resolvated in a dielectric continuum using the Generalised Born model of implicit solvation as implemented in gmx_MMPBSA (version 1.6.1). While the implicit-solvation model is applied, the free energy of solvation is calculated for the protein–ligand complex, as well as for the isolated protein and the isolated ligand. By adding the free-energy contributions from the direct interaction between the protein and the ligand, the BAP and PS workflows obtain the respective ABFE estimates.

**Figure 2:**
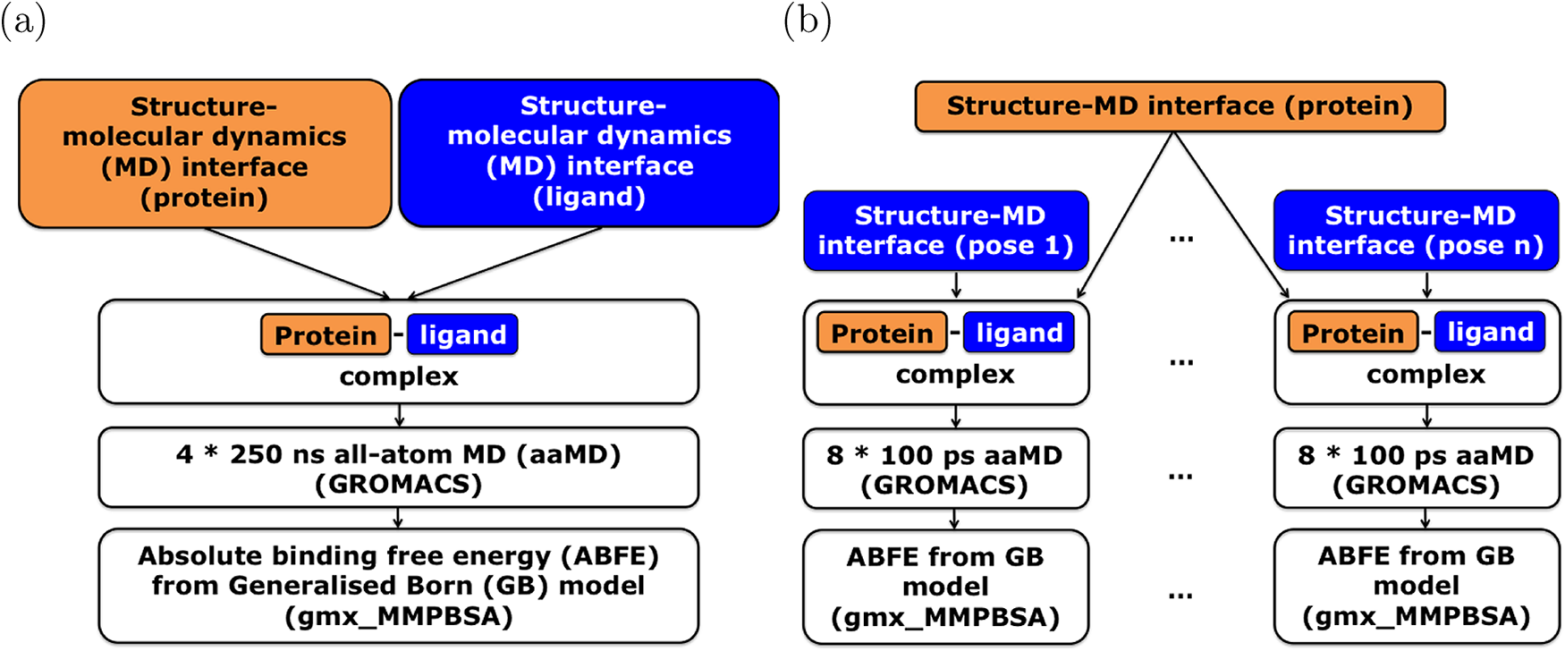
Schematic flowcharts: (a) Binding Affinity Prediction (BAP) workflow, (b) Pose Selector (PS) workflow. In both workflows, the structure-MD interface assigns force field parameters to the protein–ligand complex. The subsequent all-atom MD simulations then provide the ensemble of complex configurations to which the final implicit-solvent calculation using the Generalised Born model assigns an absolute binding free energy (ABFE). The BAP workflow computes ABFE estimates describing the binding affinity of a ligand to the target protein based on 250 ns MD simulations. In contrast, the PS workflow assigns ABFE estimates to binding poses that consider only their immediate phase space neighborhood sampled in 100 ps MD simulations.

### 4.2 The Structure-Molecular Dynamics Interface

The structure-molecular dynamics (MD) interface converts experimental structures or docking poses (proteins and ligands provided as PDB and MOL2 files, respectively) into input files for MD simulations with GROMACS, i.e., it creates a structure of the protein–ligand complex that is fit for a MD simulation and assigns AMBER force field parameters to the complex (AMBER99SB-ILDN for the protein and peptide and DNA/RNA ligands and GAFF v2.1 for all other types of ligands). The interface was written with the intention that, if an arbitrary protein–ligand complex is provided, it can determine whether the protein–ligand complex can be handled successfully by the BAP and PS workflows. Consequently, the scope of the BAP and PS workflows can be described as all complexes of ligands with soluble proteins that are accepted by the structure-MD interface. The main reasons for the interface to reject a protein–ligand complex (scientific reasons such as incorrect/incomplete structural information or complexated metal ions/non standard residues/post-translational modification for which no force field parameters were available as well as technical reasons such as crashes of the software packages involved in the interface) are summarised in Figure 3 and presented in detail in Table S1.

**Figure 3:**
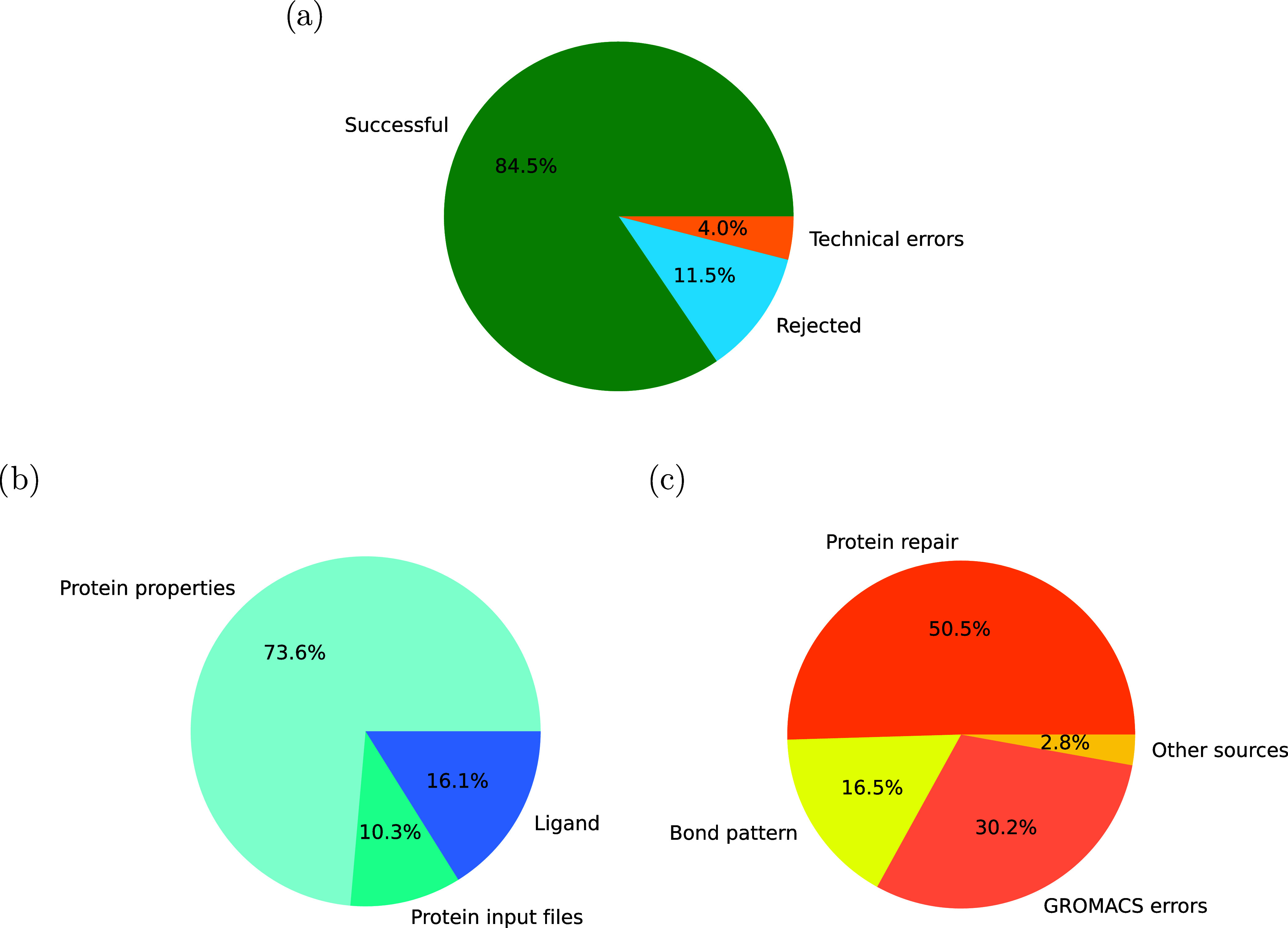
Experimental structures of the protein–ligand complexes collected in the PDBbind 2020 refined set were converted into simulation input files by the structure-MD interface: (a) distribution of successful conversions, scientific rejections and technical errors for all complexes in the PDBbind 2020 refined set, (b) distribution of scientific reasons to reject a protein–ligand complex, (c) distribution of types of technical errors. The absolute numbers are provided in Table S1.

First, the structure-MD interface classifies the protein–ligand complexes to assign them to the correct workflow branch or to reject complexes that are beyond the workflow’s scope. Moreover, it checks the complex structures for typical flaws, e.g., missing atoms, and repairs the structures if necessary and possible. In addition, the interface was equipped with a multitude of checks to catch technical errors that can occur during the different conversion steps. The inner workings of the interface and the software packages involved are described in detail in Section 1.2 of the Supporting Information, and the code is shipped as part of the workflows on GitHub. Here, we discuss the performance of the interface when it was used to convert all experimental structures collected in the PDBbind 2020 refined set^24^ into simulation input files.

Despite the significant chemical diversity of the PDBbind 2020 refined set, the structure-MD interface successfully converted 4491 out of 5316 complexes, or 84.5% (Figure 3a). 613 complexes, or 11.5%, had to be rejected for scientific reasons, and 212 complexes, or 4.0%, could not be converted due to technical errors. Although the conversion rate is already high, we will discuss the rejected complexes and the causes of technical errors in more detail to provide a notion of how many more complexes could be covered by extensions of the interface and to illustrate how carefully the interface checks the quality of the simulation input files that it produces.

451, or 73.6%, of the 613 rejected protein–ligand complexes contained proteins with properties beyond the scope of the BAP and PS workflows (Figure 3b). 81 of them were classified as membrane proteins. For the remaining 370 complexes, the cause of rejection was the need for specalised force field parameters: 178 proteins contained non standard amino acids, 191 complexes included metal ions for which the parameters are not shipped with the standard AMBER protein force fields, and one structure had to be rejected because of an unsupported co solvent. Although it was beyond the scope of this work, if the force field parameters are provided in a format that *pdb2gmx*, the GROMACS routine assigning the force field parameters, can read in, the interface can be readily extended to support these complexes, too. Another 63 complexes, or 10.3%, were rejected because the protein structure or sequence information contained flaws that could not be repaired. 50 proteins had missing atoms within 3 Å of the ligand binding site. If atoms had to be modeled within a distance of the ligand binding site that was comparable to the size of a water molecule, the modelling result would most likely determine the computed binding free energy rather than the experimental structure. For five proteins, the sequence information provided in the PDB file was inconsistent with the residue names in the coordinates of the experimental structure, and for eight proteins, the sequence was partially unknown in the official FASTA file stored in the PDB. For the remaining 99 complexes, or 16.1%, the ligand topology could not be generated: to the ligands of 74 complexes, STaGE could not assign GAFF2 force field parameters, and 25 complexes included peptide ligands with missing atoms.

The remaining 212 protein–ligand complexes could not be converted due to technical errors (Figure 3c). In 35 cases, or 16.5%, the bond pattern was not preserved by the interface: for one complex, the CONECT statements were incorrectly analysed so that a covalent bond was lost during atom renumbering, and for 34 complexes, the number of disulphide bonds was changed by the interface. However, the S atoms involved in these disulphide bonds were further apart than the distance cut off that *pdb2gmx* employed for disulphide bonds. If the protein does not exhibit structural instability in the subsequent MD simulation, e.g., unfolding or separation of subunits, which can be detected by the root-mean-square deviation (RMSD) of atom positions, the loss of these disulphide bonds will most likely not affect the binding free energy estimate in a significant manner. For this reason, these 34 complexes were only marked with a note and still converted by the interface. The leading cause of technical error, 107 complexes or 50.5%, was that the protein structure could not be successfully checked and repaired. In 15 cases, the alignment that was needed to match the names of the protein chains in the structure and sequence files failed. For 49 complexes, the detection of structural gaps suffered a fatal error. And in 43 cases, the homology model of a protein with structural gaps could not be built: ProMod 3.3. reported stereochemical problems for 17 complexes, the nonmodeled parts of 16 complexes had an RMSD > 2 Å to the experimental structure after modelling, and in 10 cases, structural gaps were still observed after modelling. For 64 complexes, or 30.2%, GROMACS programs issued fatal errors (*grompp* for 57 complexes and *mdrun* for seven complexes). Subtle flaws of the structure or the force field parametrisation that were not detected previously were most likely the cause of these fatal errors. The remaining six protein–ligand complexes, or 2.8%, could not be converted into simulation input files due to other technical errors, e.g., PDB entries for the protein could not be accessed and an error 404 (page not found) was generated.

Having passed all these checks, the converted protein–ligand complexes were used as input for MD simulations. The success rate of these simulations is an additional quality measure for the structure repair performed and the force field parameters assigned by the structure-MD interface. The results are reported in the following section.

### 4.3 Molecular Dynamics Simulations

In order to assess the quality of the force field parametrisation, the trajectories of the molecular dynamics (MD) production runs for a validation set with 42 complexes were subject to a post-processing phase that analysed the stability of each protein–ligand complex. This was achieved by checking for protein unfolding, ligand dissociation and ion dissociation, using the procedures described below.

- **Protein stability** was assessed by calculating the root-mean-square displacements (RMSD) of the backbone atoms of each protein, issuing a warning if this exceeded a threshold of 4 Å, which was deemed to exceed the normal thermal fluctuations of a stable folded protein in an NVT simulation. Over the whole validation set, this threshold was breached for at least one replica in around 29 cases. These simulations were subject to a further analysis, which included, for example, a root-mean-square fluctuation calculation of the backbone or a visual inspection of the trajectory. In all the suspect cases, it was observed that the high RMSD values were due to the intrinsic flexibility of the protein structure, rather than because of unfolding events. For example, the structure often contained highly flexible components such as disordered loops, or in other cases, domains linked in hinge-link arrangements caused large RMSD fluctuations. In no simulation was it observed that a protein actually showed signs of being structurally unstable.
- **Ligand dissociation** The stability of the ligand - protein complex was measured by calculating the center-of-mass distance between the protein and the ligand: if it increased by more than 15 Å, the ligand was considered to have dissociated from the complex (or at least partially). For this check, 9 of the 42 complexes showed signs of ligand dissociation in at least one replica. Further investigation by visual inspection confirmed that, in all these cases, the ligand had dissociated.
- **Ion dissociation** For this check, if the RMSD of any coordinated ion after fitting to the protein was more than 10 Å, the ion was considered to be not attached to a flexible side chain anymore and instead to have left its coordination site. No such ions were present in the validation set.

After analyzing the results from the runs of the validation set, we concluded that the procedure was sound and we could start the main production phase on the whole PDBbind 2020 refined set. However, during the large-scale production phase of the workflow, not all the protein–ligand complexes could be successfully equilibrated, simulated, or analysed. In particular, during the Binding Affinity Prediction (BAP) workflow, of the 4491 protein–ligand complexes for which simulation input files could be generated, 4021 were successfully equilibrated, simulated, postprocessed and analysed. Similarly, cosidering the Pose Selector workflow, the total number of complexes that could successfully arrive to the final analysis stage was 4033.

### 4.4 Protocol Validation for the Implicit-Solvent Calculations

#### 4.4.1 The Validation Set Used to Select the Implicit-Solvation Model and Its Parameters

The Binding Affinity Prediction (BAP) and Pose Selector (PS) workflows aim at providing ABFE estimates that support the development and refinement of docking scoring functions used in drug discovery. For example, the recently developed spatio temporal models Timenucy (Long-term Recurrent Convolutional Network) and Videonucy (Long-term Recurrent Convolutional Network) [3], capable of processing 4D data by using MD simulations as input, outperformed in both regression and ranking tasks on the PDBbind core set the state-of-the-art Pafnucy model (Convolutional Neural Network) trained on 3D data only. In designing the BAP workflow, we took into consideration that many protein targets relevant to drug discovery undergo conformational changes in solution that may affect the binding pocket hosting the drug candidate. Therefore, it is critical to sample the distinct protein conformations in the molecular dynamics (MD) simulations such that they are analysed by the implicit-solvation model and included in the computed ABFE estimate. We started off with 1 μs-long MD trajectories to validate the parameter choices for the implicit-solvation model, and the large RMSD values observed during the validation of the MD simulations showed that the long MD simulations indeed sampled distinct protein conformations, without a direct comparison to experimental structural data it cannot be unequivocally established whether all sampled conformations are biologically relevant, and some deviations may also arise from force-field limitations or missing structural elements. However, 1 μs-long MD trajectories for all complexes in the PDBbind 2020 refined set were not affordable with the already significant computational resources awarded to the research project. Therefore, after validating the parameters of the implicit-solvation model on MD trajectories with extensive configurational sampling under the Single Trajectory Protocol (STP), we assessed how the correlation between experimental and computed ABFEs depends on the trajectory length to identify the best trade-off between sufficiently extensive configurational sampling and available computational resources. Following this strategy, the number of complexes that could be included in the validation set was limited to well below 100 complexes. Because this number is too small to obtain a representative subset of the PDBbind 2020 refined set with its 5316 complexes by random selection, we decided to select complexes that represent the protein families that contain most of the relevant drug targets.

The protein families identified in this study - including kinases (e.g., CDK2, ABL1),^82,83^ ubiquitin ligases (MDM2, XIAP),^84,85^ proteases (MMP12, plasminogen),^86,87^ isomerases (PIN1, FKBP5)^88,89^ and bacterial adhesion proteins (FimH)^90^ - represent high-value therapeutic targets with established and emerging roles in human disease. Kinases remain the most successfully drugged family, with clinical successes like imatinib (ABL1 inhibitor) for CML,^82^ although selectivity challenges persist.^83^ Ubiquitin ligases offer promising avenues for targeted protein degradation via PROTAC technology,^84^ while proteases require more selective inhibitors to avoid the off-target effects that plagued early MMP inhibitors.^86,87^ Isomerases present untapped potential for modulating protein folding in neurological disorders,^88,89^ and bacterial adhesins enable narrow-spectrum anti-infective strategies that preserve the microbiome.^90^ Future development should focus on allosteric inhibitors,^83^ structure-guided design^86^ and combination therapies to address resistance across these target classes.

Using this approach, we also ensure that our validation set spans the binding-affinity range observed in PDBbind 2020 (Table S2). Specifically, our subset contains 63 complexes used to calibrate the method. Among them, 47 complexes span a *K*_d_ range that includes 2741 of 2783 (98.5%) complexes in the PDBbind 2020 refined set. The remaining 16 complexes span a *K*_i_ range that includes 2310 of 2533 (91.2%) complexes in the same dataset.

#### 4.4.2 Identifying the Implicit-Solvation Model to Rank Ligand Binding Poses

The Pose Selector (PS) workflow assigns absolute binding free energy (ABFE) estimates to different ligand binding poses. In this context, implicit-solvation models proved useful in identifying promising drug candidates and their most stable binding poses. For example, it was shown on a dataset of 98 chemically diverse protein–ligand complexes that both MM/GBSA and MM/PBSA predict binding poses well, estimate binding free energies accurately and are good at re scoring the top hit poses produced by other less accurate scoring functions.^70^ In fact, when the top five configurations are considered, the performance of MM/GBSA ranked second among the 12 scoring functions tested.

In this work, we benchmarked the following implicit-solvation methods:

- Molecular mechanics/generalised Born surface area (MM/GBSA),^60^
- Molecular mechanics/Poisson–Boltzmann surface area (MM/PBSA) with both linear and non linear partial differential equation (PDE) solvers^60^ and
- 3D reference interaction site model (3D-RISM)^91^

before setting out to calculate ABFEs for the protein–ligand complexes collected in the PDBbind 2020 refined set with our automated workflow. However, to the best of our knowledge, there exists no experimental reference values to validate an ABFE estimate for a single binding pose or an ABFE-based ranking of different binding poses of the same ligand because experimental techniques almost always measure the affinity with which a ligand binds to a protein, i.e., experimental ABFEs describe an ensemble of many ligand binding poses. In contrast, to assess the docking power of a docking scoring function that was developed or refined with the ABFE estimates provided by the PS workflow, the root-mean-square deviation (RMSD) of the top-ranking docking poses from ligand binding poses that were observed experimentally can be computed.^18^ So, while an established test procedure can measure potential improvements in the docking power of a docking scoring function owing to the PS workflow, its ABFE estimates could not be directly validated against experimental reference values. However, the key technical challenge of the PS workflow consists in computing a reliable ABFE estimate for a ligand binding pose with very limited configurational sampling. With the assumption that the ligand binding pose in the crystal structure is a good representative of the ensemble of ligand binding poses in solution, we therefore tested which implicit-solvation model shows the best correlation with experimental binding affinities if it is given only the crystal structure of the protein–ligand complex, which was prepared for molecular dynamics (MD) simulations by the structure-MD interface. The implicit-solvation model that yields the best correlation with experiment and, in that sense, is able to most accurately estimate the ABFE of the ligand binding pose from the crystal structure in the limit of no configurational sampling will compute the most accurate ABFE estimates for the ligand binding poses obtained by docking.

Therefore, we analysed the correlation between experimental Δ*G* values and computed Δ*G* values under the Single Trajectory Protocol (STP) using three distinct implicit-solvation models: MM/GBSA, non linear Poisson–Boltzmann surface area (NLPBSA) and 3D-RISM (Figure 4). 63 protein–ligand complexes from the PDBbind 2020 refined set were used to create a validation set (see Section 4.4.1). The ABFEs of the protein–ligand complexes were then estimated on the basis of the processed crystal structures. We conducted a linear regression analysis for each model to obtain the trendline with slope and intercept as well as the Pearson correlation coefficient (*r*-value).

**Figure 4:**
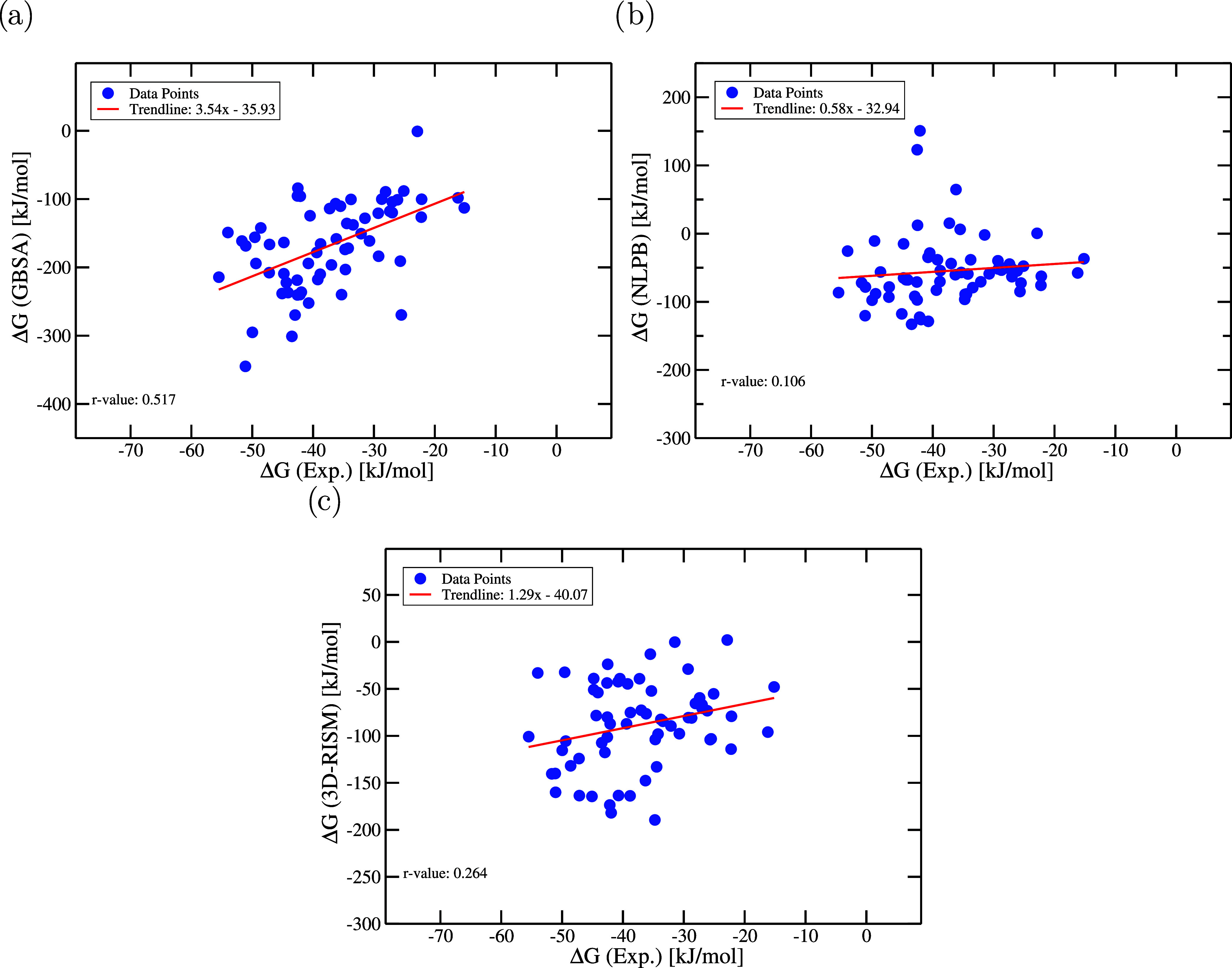
Comparison of computed Δ*G* values from different implicit-solvation models to experimental Δ*G* values for the PS Workflow: (a) Scatter plot showing the correlation between experimental Δ*G* values and Δ*G* values calculated with the Implicit Solvation models under assessment. The implicit-solvation model analysed only the crystal structure of the protein–ligand complex that had been checked and repaired by the structure-MD interface. The trendline equation is *y* = 3.54*x* – 35.93 with an *R*_*p*_-value of 0.517 (95% CI [0.309, 0.678]). (b) The non linear Poisson–Boltzmann surface area (NLPBSA) method yielded the trendline equation *y* = 0.58*x* – 32.94 with an *R*_*p*_-value of 0.106 (95% CI [−0.146, 0.345]). (c) With the MM/3D-RISM method, the trendline equation *y* = 1.29*x* – 40.07 with an *R*_*p*_-value of 0.264 (95% CI [0.017, 0.480]) was obtained. Each subplot displays data points (blue circles) and the corresponding trendline (red line) to illustrate the relationship between experimental Δ*G* and computed Δ*G* values.

During the validation of the PS workflow, the best correlation between experimental and computed ABFEs was achieved with the MM/GBSA model with a Pearson correlation of 0.517 (Figure 4a). The polar solvation contribution was evaluated having set igb = 5, corresponding to the GB-OBC2 model developed by Onufriev, Bashford and Case.^92^ This model provides improved Born radius estimates and has been widely used in MM/GBSA calculations of protein–ligand binding free energies.^60^ Details of the adopted protocol are reported in the Supporting Information. In contrast, the NLPBSA model showed the weakest correlation with experiment, achieving only an *r*-value of 0.106 (Figure 4b), not justifying the significant additional computational effort to solve the non linear PB equation. We tested the NLPBSA model because the linear version of the PB model is not guaranteed to provide accurate results for highly charged binding pockets and ligands,^64^ which can be found in many protein–ligand complexes belonging to the PDBbind 2020 refined set. MM/3D-RISM ranked second in terms of Pearson correlation with experimental values (Figure 4c), but with an *r*-value of 0.264, it still yielded a significantly weaker correlation than the MM/GBSA model. In sum, the simplest implicit-solvation model, In our experiment, MM/GBSA exhibited the best correlation with experiment for our validation set and was thus chosen for the PS workflow. Correlation was the most important criterion of this analysis because the PS workflow aims at providing a correct ranking of ligand binding poses. Therefore, the other implicit-solvation models were rejected even if they have slopes closer to 1 and intercepts closer to 0, indicating that the ABFE estimates computed with them deviate less from the experimental reference value in terms of absolute error. The exact ABFE values are listed in Table S3 of the Supporting Information.

#### 4.4.3 Identifying the Implicit-Solvation Model to Rank Protein–Ligand Complexes

The Binding Affinity Prediction (BAP) workflow aims at ranking protein–ligand complexes in terms of binding affinity to identify the most promising drug candidates for a given protein target. To this end, it assigns an ABFE estimate to an ensemble of configurations of the protein–ligand complex under study, and its ABFE estimate is a weighted average including many different ligand binding poses. Consequently, its ABFE estimates can be directly compared to experimental binding affinities, and the MD trajectory should provide a representative sample of the Boltzmann distribution. Therefore, to validate the BAP workflow, we investigated the correlation between experimental values and ABFE estimates computed under the Single Trajectory Protocol (STP) on the basis of four 1 μs-long MD trajectories per protein–ligand complex from each of which 10,001 equally spaced snapshots were extracted every 100 ps on our validation set of 63 complexes.

In this validation, the best correlation between experimental and ABFEs computed under the Single Trajectory Protocol (STP) was again achieved with the MM/GBSA model with a Pearson correlation of 0.557 (Figure 5a). Also again, the NLPBSA model showed the weakest correlation with experiment, achieving only an *r*-value of 0.161 (Figure 5b). Consequently, MM/3D-RISM ranked again second in terms of Pearson correlation with experimental values (Figure 5c), but with an *r*-value of 0.168, the correlation is significantly weaker than with the MM/GBSA model and comparable to the performance of the NLPBSA model. Because the BAP workflow aims to correctly rank protein–ligand complexes in terms of their binding affinity, correlation was again the most important criterion, and the MM/GBSA model was chosen for the BAP workflow, too. The exact ABFE values are listed in Table S4 of the Supporting Information.

**Figure 5:**
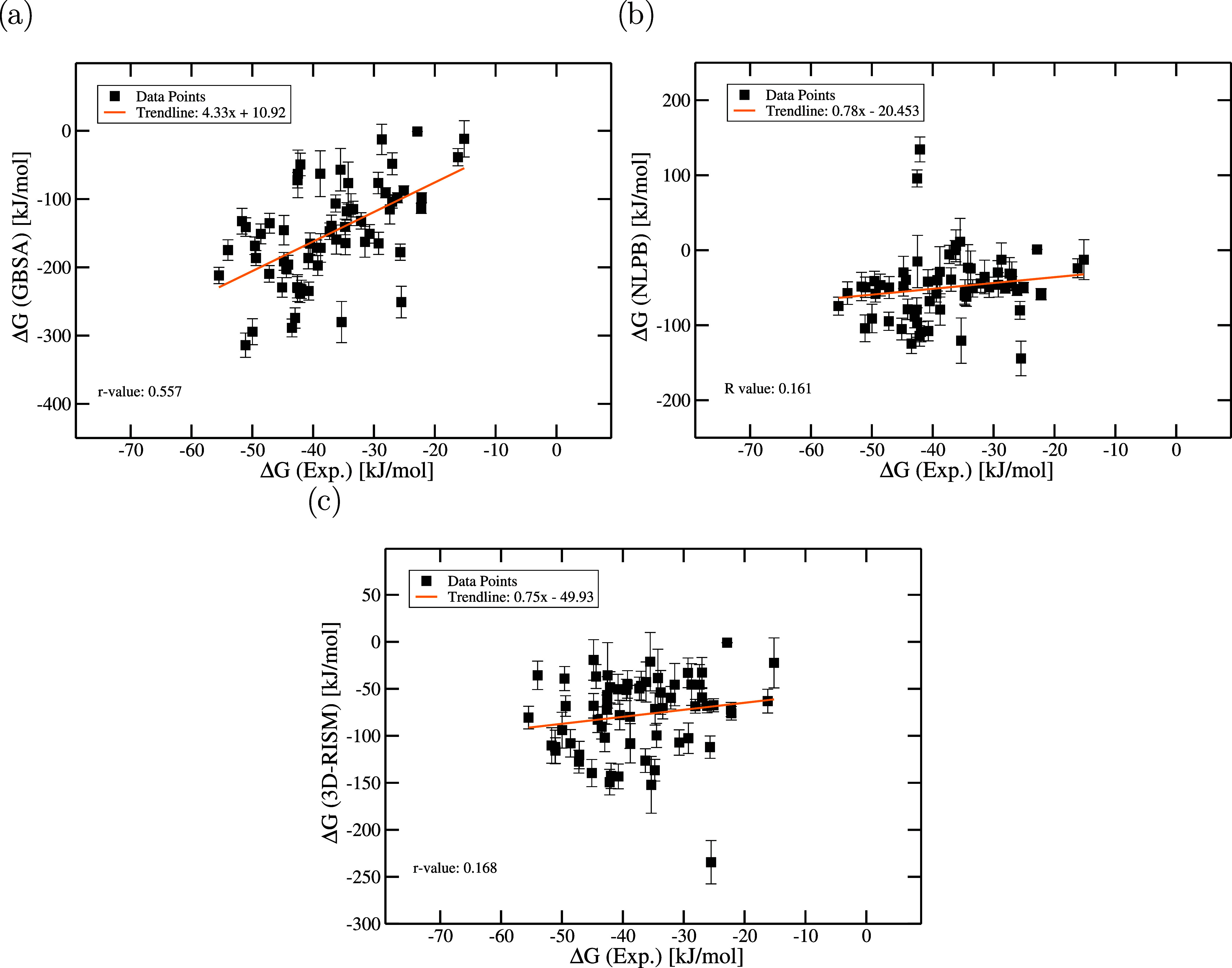
Comparison of computed Δ*G* values using different implicit-solvation models within the BAP workflow to experimental Δ*G* values: (a) Scatter plot showing the correlation between experimental Δ*G* values and Δ*G* values calculated with the generalised Born surface area (MM/GBSA) method. The trendline equation is *y* = 4.33*x* + 10.92 with an *R*_*p*_-value of 0.557 (95% CI [0.358, 0.707]). (b) The non linear Poisson–Boltzmann surface area (MM/NLPBSA) method yielded the trendline equation *y* = 0.78*x* – 20.45 with an *R*_*p*_-value of 0.161 (95% CI [−0.091, 0.392]). (c) With the MM/3D-RISM method, the trendline equation *y* = 0.75*x* – 49.93 with an *R*_*p*_-value of 0.168 (95% CI [−0.084, 0.398]) was obtained. Each subplot displays data points (black squares) and the corresponding trendline (orange line) to illustrate the relationship between experimental Δ*G* and computed Δ*G* values.

The validation results for the BAP and PS workflows suggest that the correlation between the ABFEs calculated with the selected MM/GBSA model and the experimental ABFEs exhibits an increasing trend (although not in a strictly monotonous manner) if more configurations of the protein–ligand complex under study are considered. In fact, the Pearson correlation improves from 0.517 to 0.557, with the best improvement among the trajectory lenghts assessed being recorded at 250 ns with an r-value of 0.561, as reported in Table 1. Moreover, as outlined in Theory (Section 2), the accuracy of the ABFE estimates obtained with the MM/GBSA model depends on the values of the dielectric constants of the solute and the solvent. The effects of these two parameters (configurational sampling, dielectric constants) are studied in detail in the next sections.

**Table 1:** Pearson Correlation as a Function of the Length of the Underlying MD Trajectories^a^

Trajectory length (ns)	*R*_p_-value	95% CI
0	0.517	[0.309, 0.678]
250	0.561	[0.363, 0.710]
500	0.553	[0.353, 0.704]
750	0.551	[0.351, 0.703]
1000	0.557	[0.358, 0.707]

a This table presents the *r*-values and their corresponding 95% confidence intervals (CI) obtained with different trajectory lengths, starting from the initial configuration of the trajectory and continuing in intervals of 250 ns up to 1 *μ*s. The CI width (calculated for *N* = 63) provides a measure of the precision of the correlation estimates.

#### 4.4.4 The Effect of the Dielectric Constants on the Accuracy of the ABFE Estimate

To investigate the effect of the dielectric constants on the accuracy of the ABFE estimate obtained with MM/GBSA, we assessed the Pearson correlation between experimental and ABFEs computed under the Single Trajectory Protocol (STP) for our validation set of 63 protein–ligand complexes, using two different configurational ensembles from MD simulations: starting structure only to validate the ABFE estimates to be computed with the PS workflow and 10,001 equally spaced snapshots extracted from 1 μs-long trajectories to validate the ABFE estimates to be computed with the BAP workflow. For each ensemble, we tested six combinations of values for the dielectric constants: solute dielectric constants of 1, 3, and 4 were combined with solvent dielectric constants of 78.5 and 80, the precise and a more approximate value for the dielectric constant of water.

Regardless of the values used for the dielectric constants, the Pearson correlation between experimental and computed ABFEs increases for a given combination of dielectric constants if snapshots from the 1 μs MD simulation are used for implicit-solvent calculations instead of providing the starting structure alone (Figure 6). Pearson correlation coefficients ranging from 0.398 to 0.517 were obtained if only the starting structure was analysed. With 1 μs-long MD trajectories, in contrast, correlation coefficients ranging from 0.412 to 0.557 were achieved. The corresponding correlation plots are shown in Figure S1. However, as shown in Table 1, 250 ns of MD sampling already yields a relatively high Pearson correlation coefficient (r = 0.561). This suggests that a trajectory length of 250 ns represents a reasonable compromise between analyzing only the starting structure and performing a full 1 μs simulation, while still providing a robust improvement in the correlation.

**Figure 6:**
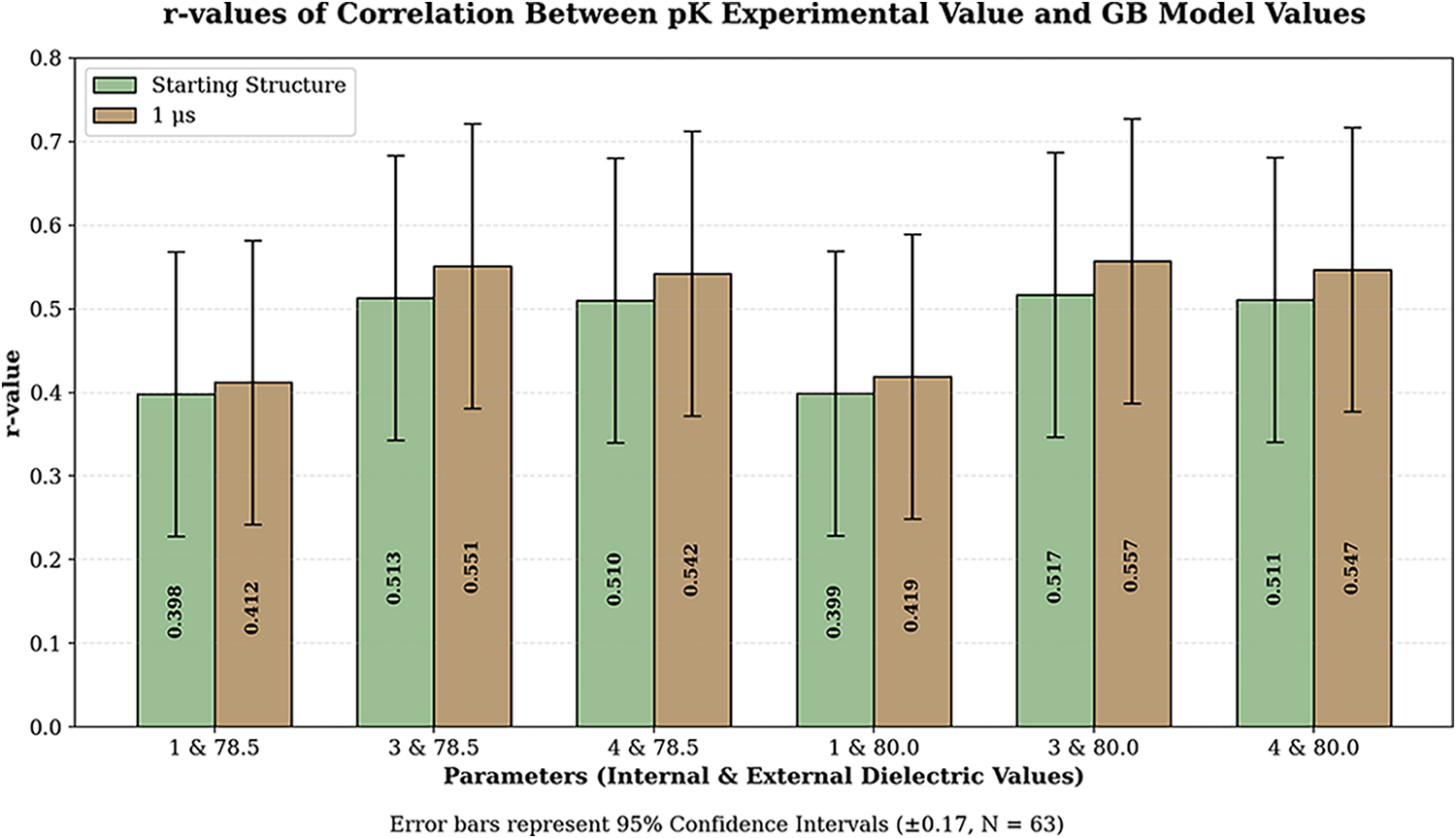
Pearson correlation between experimental Δ*G* values and Δ*G* values calculated with the generalised Born (GB) model for different solute (internal) and solvent (external) dielectric constants. The plot shows the *r*-values obtained if only the starting structure of the MD simulation (light green, PS workflow validation) or if 10,001 snapshots from 1 μs-long trajectories (brown, BAP workflow validation) were considered. Each pair of columns represents a different combination of dielectric constants. 95% Confidence Intervals (average width of ±0.17 for *N* = 63) are included.

Considering the effect of the dielectric constants (Figure 6), differences in the Pearson correlation with experiment between ABFEs computed with a solvent dielectric constant of 78.5 and ABFEs computed with a solvent dielectric constant of 80 but otherwise identical settings are minimal although slightly higher correlations were achieved with a solvent dielectric constant of 80. In contrast, the impact of the solute dielectric constant is more noticeable. With a solute dielectric constant of 1, the lowest correlation coefficients were obtained: 0.398 (starting structure) and 0.412 (1 μs-long MD trajectories) with a solvent dielectric constant of 78.5 and 0.399 (starting structure) and 0.419 (1 μs-long MD trajectories) with a solvent dielectric constant of 80. The correlation coefficients obtained with solute dielectric constants of 3 and 4 were similar if the same amount of different configurations of the protein–ligand complex was provided. With a solute dielectric constant of 3, the coefficients were 0.513 (starting structure) and 0.551 (1 μs-long MD trajectories) with a solvent dielectric constant of 78.5 and 0.517 (starting structure) and 0.557 (1 μs-long MD trajectories) with a solvent dielectric constant of 80. With a solute dielectric constant of 4, the coefficients were 0.510 (starting structure) and 0.542 (1 μs-long MD trajectories) with a solvent dielectric constant of 78.5 and 0.511 (starting structure) and 0.547 (1 μs-long MD trajectories) with a solvent dielectric constant of 80. These correlation coefficients are consistently higher than the correlations achieved with a solute dielectric constant of 1, which corresponds to vacuum or the limit of no screening of electrostatics. The higher solute dielectric constant considers that charged and polar protein residues as well as ligands with (partial) charges screen electrostatic interactions more efficiently than vacuum, without dramatically overestimating the screening by apolar moieties in the protein–ligand complex.

Because the best Pearson correlation coefficients for 1 μs-long MD trajectories and for the starting structure only (0.557 and 0.517, respectively) were achieved with a solute dielectric constant of 3 and a solvent dielectric constant of 80, these values were used in the implicit-solvent calculations of both the BAP and PS workflows. However, to validate the values of the dielectric constants used in the BAP workflow, 1 μs-long MD simulations were employed, requiring significant computational resources for each protein–ligand complex. In the following section, we therefore analyze how the Pearson correlation between experimental and ABFEs computed under the Single Trajectory Protocol (STP) changes as a function of trajectory length to save computational resources without sacrificing correlation.

#### 4.4.5 Convergence of the Pearson Correlation

After having justified our choice to set the solute and solvent dielectric constant to 3 and 80, respectively, we investigated the effect of configurational sampling on the Pearson correlation between experimental binding affinities and ABFE estimates computed with MM/GBSA. Specifically, we examined how the length of the MD trajectory (from starting structure to 1 μs) influences the correlation coefficients (*r*-values) between experimental and computed Δ*G* values. Extensive configurational sampling in the MD simulations could improve the quality of the ABFE estimates in two ways. First, the precision of the ABFE estimate increases as more configurations of the protein–ligand complex are analysed. Second, slow motions of the protein–ligand complex are more likely to be sampled as the simulated time increases. Consequently, new protein conformations and ligand binding poses are observed, the trajectory becomes a more representative sample of the Boltzmann distribution, and the accuracy of the ABFE estimate can be improved. For our validation set with 63 protein–ligand complexes, Pearson correlation coefficients were calculated using only the starting structure or the first 250 ns, 500 or 750 ns of the MD trajectory or the full 1 μs-long MD trajectory. The resulting *r*-values, representing the correlation between the experimental Δ*G* values and the Δ*G* values obtained with the MM/GBSA model, are shown in Table 1. The underlying ABFE estimates are listed in Table S5 of the Supporting Information.

Starting from 0.517 if only the starting structure of the MD trajectory is used, the Pearson correlation coefficient increases to 0.561 if the first 250 ns of the MD trajectory are included in the ABFE estimate (Table 1). If the first 500 ns of the MD trajectory are analysed, the Pearson correlation coefficient slightly decreases to 0.553 and remains stable at 0.551 if the first 750 ns of the MD trajectory are considered. For the full MD trajectory (1 μs), the Pearson correlation coefficient again slightly increases to 0.557. In sum, the correlation between experimental and computed ABFEs improves as the length of the MD trajectory is extended. However, no improvements were observed beyond 250 ns. Therefore, we limited the length of the MD trajectories of the BAP workflow to 250 ns.

The implicit-solvation models MM/GBSA and MM/PBSA were used before with the solute dielectric constant set to 4 to compute ABFEs for more than 1800 complexes from the PDBbind dataset.^26^ The best Pearson correlation coefficient achieved with MM/GBSA was *r* = 0.579 ± 0.002 when the experimentally determined crystal structures were provided to MM/GBSA. In contrast, the best overall Pearson correlation coefficient for MM/PBSA was *r* = 0.491 ± 0.003 based on 1 ns-long MD simulations, while the same indicator based on crystal structures was *r* = 0.412 ± 0.003, indicating that MD simulations are definitely needed to improve the quality of MM/PBSA results. So, the best Pearson correlation coefficient reported for MM/GBSA (*r* = 0.579 ± 0.002) on a large-scale and chemically diverse dataset was very close to the correlation coefficients achieved with the MM/GBSA model on our representative validation set and with our selection of model parameters (*r* = 0.557 and *r* = 0.517 for the BAP and PS workflows, respectively). These numbers allowed us to scale up the workflows and process protein–ligand complexes from the entire PDBbind refined set.

An approach that surpassed the Pearson correlation limit of *r* = 0.6 within the single-trajectory protocol framework, without accounting for entropy effects, involves the use of a variable dielectric GB model (VSGB 2.0).^73^ It was tested on a dataset of 855 complexes from the PDBbind set. The MM/GBSA model equipped with the VSGB 2.0 module achieved a remarkable Pearson correlation coefficient *r* = 0.79,^75^ but the model is proprietary, and it was beyond the scope of this work to develop a software tool for the automated, tailored assignment of variable dielectric constants.

### 4.5 Correlation between Computed Binding Free Energies and Experimental Values on the Large-Scale Dataset

Given the large scale of the datasets produced, we aimed at a constant, unified set of parameters for the implicit-solvent calculations that, when applied to all protein–ligand complexes in the dataset without further optimisation, robustly yields ABFE estimates that correlate well with experiment. On a validation set of 63 protein–ligand complexes from the PDBbind 2020 refined set, the conceptually simplest model, the generalised Born (GB) model, exhibited the best correlation with experimental binding affinities, with Pearson correlation coefficients of *r* = 0.557 and *r* = 0.517 in the BAP and PS validation, respectively. This level of correlation is at least competitive with existing docking scoring functions and implicit-solvent calculations carried out on the PDBbind 2020 refined set,^26^ and it is at least competitive with the correlation with experiment that expensive all-atom free-energy methods achieve on small datasets.^45^

Figure 7 depicts the correlation between computed ABFE estimates (kJ/mol) and experimental binding affinities (p*K*_i_) on the PDBbind 2020 refined set for all complexes, complexes with metals, and complexes without metals splits. Only complexes for which the BAP protocol provided a converged ABFE ≤ 0 estimate were included in each set. The following values for Pearson correlation coefficient *R*_*p*_ were obtained: (1) on all complexes (*R*_*p*_ = 0.437; 95% CI [0.408, 0.465], *p* < 0.0001); (2) on complexes with metals (*R*_*p*_ = 0.267; 95% CI [0.208, 0.364], *p* < 0.0001); and (3) on complexes without metals (*R*_*p*_ = 0.518; 95% CI [0.488, 0.546], *p* < 0.0001).

**Figure 7:**
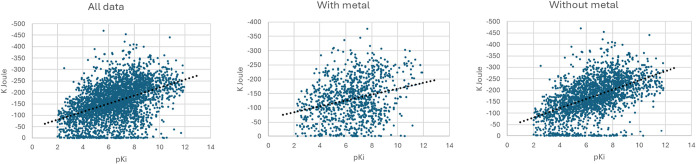
Plots showing the correlation between experimental binding affinity (p*K*_i_) and predicted binding energy (kJ/mol). The left panel includes all complexes, the middle panel shows only metal-containing complexes, and the right panel excludes all metal-containing complexes. The apparent concentration of complexes settling on the *x*-axis is a figure scaling effect due to weak binders.

Similar results were obtained for Spearman’s rank correlation coefficients, which settle at (1) *R*_*s*_ = 0.406; 95% CI [0.377, 0.435] on all complexes, (2) *R*_*s*_ = 0,236; 95% CI [0.176, 0.294] on complexes with metals and (3) *R*_*s*_ = 0,555; 95% CI [0.526, 0.583] on complexes without metals.

The lower accuracy in predicted ABFEs for metal containing complexes was expected since the MM/GBSA model is unable to accurately represent the solvation structure around the metal ion,^74^ mainly because it does not properly take into account long-range effects (like dispersion) and directional hydrogen bonds, which are known to influence the stability of the metal–ligand complex. Furthermore, the lack of accurate GB parameters for metal ions^70^ and the need for polarizable force fields to properly handle metals^93^ explain the scarcity of dedicated assessments as well as the standard procedure of removing metal containing complexes from test datasets used to evaluate MM/GBSA performances.^26,75^

Furthermore, MM/GBSA has been reported to have lower performances in predicting solvation energies for complexes including ligands with non zero formal charge.^26^

For this reason, we assessed the accuracy of the converged BAP ABFE estimates on complexes including ligands with a formal charge equal to zero and on complexes including ligands with a formal charge different from zero. Such datasets are made up of 1642 and 1799 complexes, respectively. The following values for Pearson correlation coefficient *R*_*p*_ were obtained: (1) on complexes with a ligand having formal charge equal to zero (*R*_*p*_ = 0.481; 95% CI [0.44, 0.52], *p* < 0.0001); and (2) on complexes with a ligand having formal charge different from zero (*R*_*p*_ = 0.43; 95% CI [0.391, 0.467], *p* < 0.0001).

### 4.6 Large-Scale Free-Energy Datasets As Training Data for Machine Learning-Based Scoring Functions

Having invested significant computational resources in generating the datasets, we finally explored whether the BAP dataset could support downstream machine-learning applications. To this end, we considered GenScore,^94^ a generalised protein–ligand scoring framework based on a joint residue-based (pocket) and atom-based (ligand) graph representation, graph transformation layers, and a mixture density network for binding-score prediction. The selected ML model has been shown to outperform all other models according to the CASF benchmark.^95^ We emphasize that this analysis is intended only as a preliminary proof-of-concept to illustrate the potential utility of the generated dataset, rather than as a comprehensive benchmark of ML model performance or transferability across protein families. The main idea is to explore whether the datasets generated can support this type of downstream application.

We retrained the model using the BAP dataset, including only those protein–ligand complexes to which the BAP workflow assigned a negative ABFE estimate at a 99% significance level, thus removing the complexes with positive ABFE estimates. To have a fair comparison, we also retrained the model using the experimental binding affinities reported in the PDBbind 2020 tables^24^ as labels for the same complexes included in the BAP dataset, in addition to considering the original version of the model, which was trained on the full PDBbind 2020 refined dataset.^94^ Table 2 reports the performance of the three models in terms of scoring, ranking, docking, and screening power, as typically done in scoring-function evaluations according to CASF.^95^ CASF defines scoring power as how closely a scoring function’s predicted scores correlate with experimental binding affinities; ranking power as how well it orders multiple ligands for the same target by affinity; docking power as whether the top-ranked pose reproduces the crystallographic one within a small RMSD threshold; and screening power as how effectively it separates actives from decoys across targets.

**Table 2:** Comparison of the GenScore Models Trained Using Three Different Training Sets^a^

Training Dataset		Power Metric			
Label	Complexes	Scoring	Ranking	Docking (2A)	Screening (1%)
Computed ABFEs	BAP	0.72	0.62	63.9	1.24
Experimental Affinity	BAP	0.69	0.63	54.0	1.19
Experimental Affinity	PDBbind	0.69	0.66	88.8	21.41

a Their performance was evaluated with the power metrics employed in the CASF benchmark.

When we compare the models retrained on the protein–ligand complexes contained in the BAP dataset, we notice that their performance is similar. This indicates that, on the same set of complexes, the information learned from the BAP-derived labels is comparable to that obtained from the corresponding experimental values. However, when the training is carried out on the entire PDBbind 2020 refined set, performance improves substantially, especially for docking and screening power. While this trend is qualitatively expected as the training set grows, in this case, the effect is particularly marked because the smaller retrained models operate in a low-data regime relative to the complexity of the underlying architecture. In other words, the issue is not merely the approximate two/3-fold difference in dataset size, but the fact that the BAP-only training set is close to, or below, the minimum sample size required for stable parameter estimation and generalization of the used model. Consistent with this interpretation, we observed similarly degraded performance when the model was retrained using only the same samples used for the BAP, but drawn from the original dataset. This suggests that the loss in docking and screening power mainly reflects insufficient training-set size relative to model complexity, rather than an intrinsic limitation of the BAP-derived labels. For the same reason, we expect the model trained with ABFE estimates obtained from the BAP workflow to improve as the training set is expanded with carefully selected additional instances, for example, through active-learning strategies.^96^

## 5 Conclusions

Large-scale datasets of consistent binding free-energy estimates for a chemically diverse collection of protein–ligand complexes are a valuable tool to systematically assess and then improve the performance of docking scoring functions or computational free-energy techniques. In this work, we used molecular dynamics (MD) simulations in combination with implicit-solvation models to obtain sets of consistent absolute binding free energy (ABFE) estimates for protein–ligand complexes from the PDBbind 2020 database and assessed the accuracy of the computed ABFE estimates by determining their correlation with experimental binding affinities if available. On the one hand, we calculated ABFE estimates describing how stably the ligand is bound to the protein pocket, judging the ensemble of binding poses of the ligand in the binding pocket; on the other hand, we computed ABFE estimates describing how stable a single binding pose is. The computational protocols that we employed are made available as the Binding Affinity Prediction (BAP) and Pose Selector (PS) workflows, respectively. All workflow stages, the creation of simulation input files from structure files in the structure-MD interface, the MD simulations and the subsequent implicit-solvent calculations, were carefully validated before running the workflows at large-scale. The BAP workflow was run on the PDBbind 2020 refined set and yielded MD trajectories and ABFE estimates for >4000 protein–ligand complexes, while the PS workflow was executed on >4000 complexes from the full PDBbind 2020 database and for >800,000 binding poses of these complexes, including both experimental binding poses and docking poses. Thus, the BAP and PS workflows can be and were executed at significantly larger scale than other workflows that combine molecular dynamics and implicit-solvent calculations.^97,98^ The larger scale was achieved thanks to the structure-MD interface, which fully automates the generation of simulation input files and performs more thorough quality checks and repair operations on the user-provided protein–ligand complexes than other workflows, and thanks to the integration with the meta-scheduler HyperQueue, with which millions of simulation jobs can be submitted in a fully automated fashion on HPC resources.

The BAP and PS workflows have produced two public, large-scale datasets that pair structural data with ABFE estimates that allow ranking protein–ligand complexes as well as ligand binding poses, thus providing training data for the development and improvement of docking scoring functions. As the workflow scripts are publicly available and free to use, these datasets can be readily extended, also enabling studies on the best strategies to expand structural data for machine learning, e.g., by rather extending the number of protein–ligand complexes in the dataset or the amount of configurational sampling per complex. Moreover, the workflows can be used to develop and refine MD-based free-energy methods. Thanks to the modular design of the BAP and PS workflows, these new or modified free-energy methods can be easily integrated into the workflows and tested at large-scale, simply by replacing the script performing the implicit-solvent calculation. In particular, if the BAP and PS datasets are fused with data from related projects, the two workflows are thus valuable tools to foster the development and refinement of docking scoring functions and free-energy methods at large-scale on a dataset representing the chemical diversity of drug candidates for soluble proteins.

## Supplementary Material



## Data Availability

The workflow scripts are available on GitHub.[Fn fn1] The MD simulation input files for the BAP workflow, the docking poses produced with LiGen for the full PDBbind 2020 database, the MD simulation input structures for the PS workflow, as well as all ABFE estimates, were published on Zenodo.[Fn fn2] On top of that, we are uploading the BAP MD trajectories to the CINECA node of the Molecular Dynamics Database (MDDB) project, where they can be found by using the search term “ligate”.[Fn fn3]

## Notes

Technical details on how structure files for protein–ligand complexes were prepared for MD simulations, and detailed parameters for docking, MD simulations, and implicit-solvent calculations (PDF)
