# Supplementary material for: Molecular Dynamics Workflows to Compute Large-Scale Sets of Absolute Binding Free Energies Aiding Drug Candidate and Binding Pose Selection
Source: J Chem Theory Comput. 2026 May 13;22(10):5122–42. doi: 10.1021/acs.jctc.5c02127 (PMC13217541; doi:10.1021/acs.jctc.5c02127)
Supplement: Supplementary file 1 [file ct5c02127_si_001.pdf]

Molecular Dynamics Workflows to Compute Large-Scale Sets of Absolute Binding Free Energies Aiding Drug Candidate and Binding Pose Selection

Sebastian Wingbermühle,[†,‡] Akash Deep Biswas,[†,¶] Domenico Bonanni,[*,†,¶,§,||] Tatiana Shugaeva,[‡] Davide Gadioli,[⊥] Jakub Beránek,[#] Giorgia Frumenzio,[@] Lara Querciagrossa,[@] Andrea Piserchia,[@] Gianmarco Accordi,[⊥] Filippo Lunghini,[¶] Carmine Talarico,[¶] Andrew Emerson,[@] Jan Martinovič,[#] Andrea Rosario Beccari,[¶] Gianluca Palermo,[⊥] and Erik Lindahl[‡]

†S.W., A.D.B. and D.B. contributed equally to this work.

‡Dept. Applied Physics, Science for Life Laboratory, KTH Royal Institute of Technology, Solna, 17165, Sweden

¶EXSCALATE, Dompé Farmaceutici S.p.A., Naples, 80131, Italy

§Department of Physical and Chemical Sciences, Università degli Studi dell'Aquila, L'Aquila, 67100, Italy

||Data Science and Computation Facility, Istituto Italiano di Tecnologia, Genova, 16163, Italy

⊥Dept. of Electronics, Information and Bioengineering, Politecnico di Milano, Milan, 20133, Italy

#IT4Innovations, VSB – Technical University of Ostrava, Ostrava-Poruba, 70800, Czech Republic

@HPC Department, CINECA, Bologna, 40033, Italy

E-mail: domenico.bonanni@iit.it

1 Detailed Methods

1.1 Preparation of Docking Structures

The full PDBbind 2020 release,[1] including 19,433 protein–ligand complexes, was downloaded from the official repository (`http://www.pdbbind.org.cn/`). All protein and ligand files were standardised, using three complementary utilities:

1. Protein Preparation Wizard (Schrödinger),[2] which was used to add hydrogen atoms missing in the crystal structure, remove crystallographic waters beyond 5 Å from hetero groups, i.e. chemical moieties that are neither pure hydrocarbons nor standard amino acids, to assign protonation states, optimise hydrogen bonding networks and perform a restrained energy minimisation;

2. PDBFixer[3] to add missing and repair partially resolved residues, e.g. by inserting absent side chains, and resolve atom clashes and valence errors and

3. LigPrep (Schrödinger)[4] to add missing hydrogens, assign protonation states and perform a single-conformation energy minimisation.

This three-step protocol eliminated common chemical issues such as atom clashes, missing or incomplete protein residues and non-physiological valence states, yielding a fully standardised input set.

For the Pose Selector (PS) workflow, we generated de novo docking poses with LiGen[5] for the full PDBbind dataset. Up to 256 poses per ligand were produced using the software's default parameters. Before docking, the crystallographic ligand was rearranged to maximise its internal volume (i.e., the sum of all interatomic distances) to avoid any bias from the experimental binding pose. The ligand was then re-docked into its binding pocket, considering the centroid of the experimental pose.

The LiGen docking algorithm follows a multi-restart gradient-descent method (by default, 256 restarts in the present work). For the initial pose generation, at each restart, the rotatable

bonds of the ligand are rotated to maximise diversity in conformational space. After that, a rigid-body alignment step is done. The ligand (treated as rigid) is roto-translated to best fit the binding cavity. Finally, starting from the previous pose, a flexible refinement is implemented by optimizing the rotatable bond of the ligand inside the binding site. A geometric score combined with physical-chemical properties drives a local gradient descent.

All input structures generated in this way are available on Zenodo:(`https://doi.org/10.5281/zenodo.11397017` and `https://doi.org/10.5281/zenodo.11397486`).

1.2 Structure-Molecular Dynamics Interface

The structure-MD interface first classified, checked and, if necessary, repaired the protein structure provided as input. To this end, it generated a FASTA file containing the sequence of amino acids resolved in the input structure and rejected the protein if non-standard amino acids were found in the structure. The resulting sequence was checked for membrane-spanning or membrane-inserting regions with TMbed 1.0.0,[6] and if they were detected, the protein was rejected. Next, the canonical FASTA file containing the full protein sequence was downloaded from the PDB database. If the canonical FASTA file was not available or contained unknown residues indicated by the letter "X", the protein was rejected. Subsequently, structural gaps in the input structure were detected with pdb-tools 2.5.0.[7] Two Python scripts from the pdb-tools package were used: pdb_delhetatm, which removed all HETATM records in the PDB file as a necessary preparation step for gap detection, and pdb_gap, which detected gaps by using both the distances between atoms in consecutive residues and discontinuous residue numbering. Structural gap coordinates were used to divide the sequence into regions present in the structure file and the missing regions. Gap start and end coordinates were checked to handle cases with special residue numbering, such as antibody hypervariable regions with letter-based residue naming. When special numbering resulted in multiple residues having the same number, causing ambiguity in gap start or end coordinates, missing regions were marked broadly to ensure no residues were missed.

For all residues identified as missing, a "." was inserted as placeholder in the sequence of amino acids resolved in the input structure. Next, the protein chains in the input structure and in the canonical FASTA file were matched on the basis of alignment scores calculated with biopython 1.81,[8] bypassing inconsistent chain naming in structure and sequence files. If gaps had to be filled, the placeholders were replaced with the actual sequence from the canonical FASTA file using Python's string search before it was finally checked whether the resulting protein sequence to be simulated was a sub-string of the full sequence in the canonical FASTA file, enabling the interface to detect alignment errors or mismatches between the input structure and the canonical protein sequence if there were no structural gaps. With this strategy, the structure-MD interface only filled gaps in the middle of a chain, whereas terminal deletions were not modelled. If structural gaps were present, a homology model for the protein sequence to be simulated was built with ProMod 3.3 as shipped with Open Structure 2.4,[9,10] using the input structure as template and then closing the structural gaps as well as modelling missing side chains and backbone atoms of partially resolved residues. For later checks, it was documented which atoms and residues were modelled and whether partially resolved terminal residues were omitted in the homology model. Water molecules and ions for which coordinates were provided in the input structure file were added back to the resulting structural model of the protein. Moreover, CONECT statements indicating disulphide bonds and bonds with coordinated metal ions were updated. With the above setup, the input coordinates could be changed if side chains needed to be rebuilt differently, e.g. to remove steric clashes after modelling or to minimise the energy of the resulting model. To only simulate homology models that were very similar or identical to the resolved parts of the input structure, the root mean square deviation (RMSD) between the input structure and the same residues in the homology model was calculated with rmsd 1.5.1 (http://github.com/charnley/rmsd),[11,12] and the protein was rejected if this RMSD exceeded 2 Å.

Next, the structure-MD interface prepared the topology, i.e. it assigned force field pa-

rameters to both the protein and the ligand. First, the interface rejected protein-ligand complexes containing coordinated metal ions and co-solvents that were not supported by the AMBER force fields used, while modifying the residue names of supported ions to ensure that they meet the naming conventions of the force fields. Next, the protein topology was generated using pdb2gmx as implemented in GROMACS 2023.2,[13–19] assigning the parameters of the AMBER99SB-ILDN protein force field[20–22] and the TIP3P water model.[23] If disulphide bonds were present, all protein subunits were merged into one topology; otherwise, they were parameterised in separate topologies. If long bonds were reported during the generation of the protein topology, structural gaps were still present after the homology modelling step, and the protein-ligand complex was rejected. Moreover, the numbers of residues and atoms added in the homology model were updated to be consistent with the numbering used by pdb2gmx.

In contrast to the Binding Affinity Prediction (BAP) workflow, the Pose Selector (PS) workflow simulated several binding poses per ligand. To this end, the structure-MD interface of the PS workflow extracted the different binding poses of the ligand from the input MOL2 file and prepared and looped over sub-folders for the different simulation starting structures. Otherwise, the structure-MD interfaces of the BAP and PS workflow are identical if not explicitly stated otherwise. As first step of the ligand parameterisation, the ligand was classified as peptide, DNA/RNA or small organic molecule based on the residue name. Combinations, e.g. amino acids bound to nucleotides, were classified as small organic molecules, too. To employ the AMBER99SB-ILDN force field for peptides and DNA/RNA, these ligands were parameterised with pdb2gmx. Warnings about long bonds indicated that atoms of the peptide ligand were not resolved in the structure provided as workflow input, and the protein-ligand complex was rejected. To obtain a topology for the full protein-ligand complex, the structure file of the full complex was converted back to PDB format and the whole complex was parameterised again with pdb2gmx, keeping the ligand as separate chain and enforcing the protonation pattern used in the individual parameterisations of protein

and ligand. In the PS workflow, it was explicitly checked that the protonation pattern was preserved among different binding poses. In contrast, ligands classified as small organic molecules were parameterised with the GAFF 2.1 force field.[24] The parameterisation was managed by STaGE 1.0.0 (https://gitlab.com/gromacs/stage) calling ACPYPE[25,26](as provided on https://github.com/alanwilter/acpype on 21 July 2022) employing AnteChamber from AmberTools 21.[24,27] The partial charges of ligands frequently did not sum up to the correct total charge but were off by one unit after rounding, e.g. the partial charges of a ligand with a total charge of -3 summed up to -2.3. Therefore, the structure-MD interface reran the ligand parameterisation with STaGE using the other neighbouring integer charge if the first parameterisation was unsuccessful. In the above example, a total charge of -2 would be used in the first parameterisation attempt before the parametersation would be successful with a total charge of -3. As the ligands were assumed to be closed-shell molecules with a total electron spin $S = 0$, only one of the two total charges could result in the correct total electron spin and the incorrect total charge was guaranteed to cause the quantum mechanical calculation during parameterisation to fail.

After file re-arrangements to obtain one topology per protein-ligand complex and one structure file per binding pose including water molecules and coordinated ions from the input structure, two further quality checks were performed. The minimum distance between modelled protein atoms and the ligand was computed. To avoid that the result of the homology modelling step impacted the binding free-energy estimate to be computed, the binding pose was rejected if this distance was below a threshold of 3 Å, i.e. the approximate size of a water molecule. Moreover, it was checked whether the pattern of disulphide bonds indicated in the CONECT statement of the input structure file was preserved. If not, the protein-ligand complex was not discarded, but a warning was issued such that it could be inspected later on whether the different disulphide bond pattern affected protein dynamics and stability.

Having obtained a topology and input coordinates that are guaranteed to be free of the

typical flaws in experimental structures, the protein-ligand complex was placed in a rhombic dodecahedron such that the distance between the walls of the simulation cell and each atom of the protein-ligand complex amounted to at least 1.5 nm. Next, the protein-ligand complex was solvated in TIP3P water (experimentally resolved water molecules were kept) and Na and Cl ions were added as needed to obtain a neutral total charge in the simulation box. To avoid steric clashes with surrounding water molecules resulting in unsuccessful simulations, the energy of the system was minimised with steepest descent, using flexible bonds. However, to change the experimental or docking structure as little as possible, the minimisation was limited to 100 steps, and position restraints with a force constant of $1000\,\mathrm{kJ\,mol^{-1}\,nm^{-2}}$ were applied to the coordinates of the protein-ligand complex. Periodic boundary conditions were employed in all spatial directions, and non-covalent interactions were handled with the Verlet cut-off scheme. Using Particle Mesh Ewald (PME),[28] Coulomb interactions were calculated in real space up to an atom-atom distance of 1.0 nm and in reciprocal space beyond the cut-off. Van der Waals interactions were calculated up to a cut-off at 1.0 nm, while an analytical dispersion correction for energy and pressure accounted for the omitted interactions. At the cut-off, both the Coulomb and van der Waals potentials were adjusted by the potential shift modifier. The resulting output structure was used as input positions for the MD simulations of the BAP and PS workflows (initial velocities were drawn randomly with different seeds for all replicas).

1.3 Molecular Dynamics Simulations

In the BAP workflow, the system was equilibrated for 5 ns before the production simulation. Configurations were propagated with the Verlet leapfrog integrator, using a 2 fs time step. Structures, energies and log data were saved to disk every 10 ps. Periodic boundary conditions were employed in all spatial directions, and non-covalent interactions were handled with the Verlet cut-off scheme. Using PME, Coulomb interactions were calculated in real space up to an atom-atom distance of 1.0 nm and in reciprocal space beyond the cut-off.

Van der Waals interactions were calculated up to a cut-off at 1.0 nm, while an analytical dispersion correction for energy and pressure accounted for the omitted interactions. At the cut-off, both the Coulomb and van der Waals potentials were adjusted by the potential shift modifier. The v-rescale thermostat[29] kept the temperature at 298 K with a time constant of 0.1 ps, while two coupling groups for the protein-ligand complex and for water and ions were used. The isotropic C-rescale barostat[30] maintained the pressure of the full system at 1.0 bar with a time constant of 0.5 ps. Initial velocities were randomly drawn from the Boltzmann distribution at 298 K. Hydrogen bonds were constrained using LINCS,[31,32] and the positions of the heavy atoms of the protein-ligand complex were restrained with a force constant of $1000\,\mathrm{kJ\,mol^{-1}\,nm^{-2}}$. This equilibration was not performed in the PS workflow.

The parameters of the four production simulations per complex of the BAP workflow differed from the parameters of the equilibration in only three points: the length of the trajectory was increased to 250 ns, the position restraints on the protein-ligand complex were removed, and the time constant of the C-rescale barostat was increased to 2.0 ps.

For the PS workflow, the parameters of the eight production simulations per pose were almost identical to the parameters of the BAP production simulations. However, the length of an individual trajectory was limited to 100 ps, and structures, energies and log data were saved to disc every 1 ps.

After the MD simulations were complete, all water molecules and ions were removed because the implicit-solvent calculations did not support their presence in the trajectory. Moreover, configurations in which the protein-ligand complex was broken across the periodic boundary conditions were made whole, and all configurations of the protein-ligand complex in the entire trajectory were fit to the starting structure. This post-processed trajectory was used as input for the implicit-solvent calculations of the BAP and PS workflows.

1.4 Implicit-Solvent Calculations

To obtain absolute binding free energy (ABFE) estimates with the GBSA model of implicit solvation, the same force fields for the protein (AMBER99SB-ILDN) and the ligand (GAFF, v 2.1) were employed as in the MD simulations. Moreover, the ion parameters proposed by Li and Merz were selected, which are compatible with the TIP3P water model against which the protein force field was parameterised. The atom radii used by the GB model (mbondi2) were optimised for AMBER force fields. The temperature was set to 298.15 K, and all approaches to estimate the change in the configurational entropy (quasi-harmonic approximation, interaction entropy and C2 entropy) were switched off.

To calculate the solvation contribution to the ABFE of the protein-ligand complex, the modified generalised Born model 2[33] was employed, without using the analytical linearised Poisson-Boltzmann (ALPB) approximation. When the BAP and PS workflows were applied to their large-scale datasets, the dielectric constants of the solute, the protein-ligand complex, and of the solvent, water, were set to 3.0 and 80.0, respectively. The values tested during the validation of the BAP workflow are described in Section 4.4.3 of the main manuscript. The salt concentration amounted to $0.0\,\mathrm{mol\,L^{-1}}$. The surface tension was set to the default value of $0.0301\,\mathrm{kJ\,mol^{-1}\,\AA^{-2}}$, while no offset was applied to correct the value of the non-polar contribution to the solvation free energy term. The surface area used to compute the non-polar contribution to the solvation free energy term was calculated by employing the linear combination of pairwise overlaps while the radius of the probe molecule representing a water molecule was set to 1.4 Å and no offset was applied to the atomic radii. Potential energies for the configurations of the protein-ligand complex were obtained using only the classical force fields listed above.

The snapshots to be analysed with the GBSA model were extracted from the MD trajectory with GROMACS 2023.2 so that the whole trajectory file provided to gmx_MMPBSA 1.6.1 was read in. Because a reference structure of the complex was provided, chain IDs were ignored by gmx_MMPBSA. No experimental K_i values were provided. To save disk

space, the output files produced by gmx_MMPBSA were reduced to the minimum necessary to obtain the ABFE estimate, while its verbosity was set to the maximum value to have access to all contributions to the ABFE that were calculated. Because the protein-ligand complex had been extracted from explicit water with GROMACS before, the removal of ions and water molecules as implemented in gmx_MMPBSA was not used.

The input file used for the GBSA calculations is shown on the next page.

```
# General namelist variables
&general
  startframe            = 1
  endframe              = 9999999
  interval              = 1
  forcefields           = "oldff/leaprc.ff99SBildn,leaprc.gaff2"
  ions_parameters       = 1
  PBRadii               = 3
  temperature           = 298.15
  qh_entropy            = 0
  interaction_entropy   = 0
  c2_entropy            = 0
  assign_chainID        = 0
  exp_ki                = 0.0
  full_traj             = 0
  keep_files            = 0
  netcdf                = 0
  solvated_trajectory   = 0
  verbose               = 1
/

# (AMBER) Generalized-Born namelist variables
&gb
  igb                   = 5
  intdiel               = 3.0
  extdiel               = 80.0
  saltcon               = 0.0
  surften               = 0.0072
  surfoff               = 0.0
  molsurf               = 0
  msoffset              = 0.0
  probe                 = 1.4
```

```
  ifqnt                  = 0
  alpb                   = 0
/
```

To obtain absolute binding free energy (ABFE) estimates with the PBSA model of implicit solvation, the same force fields for the protein (AMBER99SB-ILDN) and the ligand (GAFF, v 2.1) were employed as in the MD simulations. Moreover, the ion parameters proposed by Li and Merz were selected, which are compatible with the TIP3P water model against which the protein force field was parameterised. The atom radii used by the PB model (mbondi2) were optimised for AMBER force fields. The temperature was set to 298.15 K, and all approaches to estimate the change in the configurational entropy (quasi-harmonic approximation, interaction entropy and C2 entropy) were switched off.

To calculate the solvation contribution to the ABFE of the protein-ligand complex, the non-linear Poisson-Boltzmann equation was solved without using the sander APBS module available in Amber. The modified ICCG solver was employed, while the maximum number of solver iterations was set to 10,000, and a single iteration was considered converged if the residual was reduced to 0.0001 times its initial value. The ratio between the longest dimension of the finite-difference grid employed by the solver and the longest dimension of the solute, the protein-ligand complex, was set to 4.0, and the resolution of the Poisson-Boltzmann grid was set to 2.0. The finite-difference grid was not extended with buffer units beyond the surface of the protein-ligand complex. If electrostatic focusing calculations were needed, at most two successive calculations were allowed, the finer grid was eight times as dense as the coarse grid used else, and the finite-difference grid was regenerated once.

Potentials at the boundary of the grid were calculated considering all grid charges, and surface charges at the dielectric boundary between the protein-ligand complex and water were not scaled before computing the electrostatic interaction with the continuum solvent. The full electrostatic potentials and energies were obtained with the particle-particle particle-mesh protocol.[34] The cut-off values for finite-difference, Coulomb and van der Waals interactions were set to 1 nm, mimicking the cut-off values used during the MD simulations. Atom pair lists were updated every step. Electrostatic forces were not calculated.

The dielectric interface between the protein-ligand complex and water was built using a

geometric approach.[35] The surface of the protein-ligand complex was determined with the solvent-excluded surface algorithm,[36] and the radius of the probe molecule representing a water molecule was set to 1.4 Å. The radii of atoms of the protein-ligand complex were read from the MD topology and used unmodified. Solvent-accessible arcs were represented with a resolution of 0.25 Å. The dielectric constants of the solute, the protein-ligand complex, and of the solvent, water, were set to 3.0 and 80.0, respectively. At the boundary between the protein-ligand complex and water, the dielectric constant was calculated as a harmonic average depending on the extent to which a grid edge was inside or outside the volume occupied by the protein-ligand complex.[37] Like the salt concentration for GBSA, the ionic strength was set to $0.0\,\mathrm{mol\,L^{-1}}$.

The non-polar contribution to the solvation free energy term consisted of a dispersion term obtained by integrating the interactions between the protein-ligand complex and water, which is modelled like a PCM continuum solvent known from quantum chemistry algorithms,[38] over the surface of the protein-ligand complex[39] as well as a cavity term proportional to the volume enclosed by the solvent-accessible surface area of the protein-ligand complex. The σ decomposition scheme was employed,[39] and the atomic solvent-accessible surface was represented by approximately 400 dots while up to 1500 arc dots were stored per atom. To compute the solvent-accessible area for the dispersion term, the radius of the water probe was set to 0.557 Å[39] and the effective water density to 1.129.[39] To obtain the volume for the cavity term, the radius of the water probe was chosen to be 1.300 Å.[39] The standard σ values were used as van der Waals radii of the atoms in the protein-ligand complex. The regression coefficient and offset for the cavity term were set to 0.0378 and -0.5692, recommended values for the σ decomposition scheme.[39]

The snapshots to be analysed with the PBSA model were extracted from the MD trajectory with GROMACS 2023.2 so that the whole trajectory file provided to gmx_MMPBSA 1.6.1 was read in. Because a reference structure of the complex was provided, chain IDs were ignored by gmx_MMPBSA. No experimental K_i values were provided. To save disk

space, the output files produced by gmx_MMPBSA were reduced to the minimum necessary to obtain the ABFE estimate, while its verbosity was set to the maximum value to have access to all contributions to the ABFE that were calculated. Because the protein-ligand complex had been extracted from explicit water with GROMACS before, the removal of ions and water molecules as implemented in gmx_MMPBSA was not used.

The input file used for the PBSA calculations is shown on the next page.

```
# General namelist variables
&general
  startframe             = 1
  endframe               = 9999999
  forcefields            = "oldff/leaprc.ff99SBildn,leaprc.gaff2"
  ions_parameters        = 1
  PBRadii                = 3
  temperature            = 298.15
  qh_entropy             = 0
  interaction_entropy    = 0
  c2_entropy             = 0
  assign_chainID         = 0
  exp_ki                 = 0.0
  full_traj              = 0
  keep_files             = 0
  netcdf                 = 0
  solvated_trajectory    = 0
  verbose                = 1
/

# (AMBER) Possion-Boltzmann namelist variables
&pb
  ipb                    = 1
  inp                    = 2
  npbopt                 = 1
  sander_apbs            = 0
  indi                   = 3.0
  exdi                   = 80.0
  smoothopt              = 1
  istrng                 = 0.0
  radiopt                = 0
  prbrad                 = 1.4
```

```
  sasopt             = 0
  arcres             = 0.25
  memopt             = 0
  solvopt            = 1
  accept             = 0.0001
  linit              = 10000
  fillratio          = 4.0
  scale              = 2.0
  nbuffer            = 0.0
  nfocus             = 2
  fscale             = 8
  npbgrid            = 1
  bcopt              = 5
  eneopt             = 1
  frcopt             = 0
  scalec             = 0
  cutfd              = 10.0
  cutnb              = 10.0
  nsnba              = 1
  decompopt          = 2
  use_rmin           = 0
  sprob              = 0.557
  vprob              = 1.3
  rhow_effect        = 1.129
  use_sav            = 1
  cavity_surften     = 0.0378
  cavity_offset      = -0.5692
  maxsph             = 400
  maxarcdot          = 1500
  npbverb            = 0
/
```

To obtain absolute binding free energy (ABFE) estimates with the 3D-RISM model of implicit solvation, the same force fields for the protein (AMBER99SB-ILDN) and the ligand (GAFF, v 2.1) were employed as in the MD simulations. Moreover, the ion parameters proposed by Li and Merz were selected, which are compatible with the TIP3P water model against which the protein force field was parameterised. The atom radii used by the 3D-RISM model (mbondi2) were optimised for AMBER force fields. The temperature was set to 298.15 K, and all approaches to estimate the change in the configurational entropy (quasi-harmonic approximation, interaction entropy and C2 entropy) were switched off.

To calculate the solvation contribution to the ABFE of the protein-ligand complex, the molecular Ornstein-Zernike equation underlying the 3D-RISM model was solved with the Kovalenko-Hirata closure approximation with a tolerance of 0.00001. No corrections to the solvation free energy based on excess chemical potential functionals (Gaussian fluctuation, PC+) were applied, but long-range asymptotic corrections were used to analytically account for the distribution of solvent molecules outside the solvation box. To calculate the long-range asymptotic correction for the direct and total correlation function, the treecode approximation was employed while the multipole acceptance criterion was set to 0.1, the Taylor series was truncated after the second-order term, and the number of grid points in each treecode leaf cluster was limited to 500. In contrast, the full Coulomb potential energy was computed as the direct sum. The tolerance of the Coulomb potential calculation in reciprocal space was set to 0.000001, one tenth of the tolerance required for the Kovalenko-Hirata closure. The minimum distance between the solute atoms and the edges of the solvation box was set to 14 Å, and a grid spacing of 0.5 Å was used in all three spatial directions inside the solvation box. By specifying a negative value for the cutoff for solute-solvent interactions, the initial cutoff for Lennard-Jones interactions was implicitly set to 14 Å, too. During the calculation, the cutoff for Lennard-Jones interactions was adjusted to the box size such that errors caused by the truncation of the Lennard-Jones potential could be analytically corrected. To obtain the solvation free energy according to the 3D-RISM model, the MDIIS

solver was employed with a step size of 0.7. The results of five previous iterations were used to predict a new solution, and the solver was re-started from the previous solution with the lowest residual stored in memory if the current residual was ten times or more larger than the lowest residual. At most, 10,000 iterations were allowed to obtain a converged solvation free energy. The initial guess for the current atom configuration of the solute was created on the basis of five previous solutions. Only the final solvation free energy provided by 3D-RISM was printed; information about polar, non-polar and entropic contributions as well as about the number of iterations required to obtain a converged solution was not considered.

The snapshots to be analysed with the 3D-RISM model were extracted from the MD trajectory with GROMACS 2023.2 so that the whole trajectory file provided to gmx_MMPBSA 1.6.1 was read in. Because a reference structure of the complex was provided, chain IDs were ignored by gmx_MMPBSA. No experimental K_i values were provided. To save disk space, the output files produced by gmx_MMPBSA were reduced to the minimum necessary to obtain the ABFE estimate, while its verbosity was set to the maximum value to have access to all contributions to the ABFE that were calculated. Because the protein-ligand complex had been extracted from explicit water with GROMACS before, the removal of ions and water molecules as implemented in gmx_MMPBSA was not used.

The input file used for the 3D-RISM calculations is shown on the next page.

```
# General namelist variables
&general
  startframe            = 1
  endframe              = 9999999
  interval              = 1
  forcefields           = "oldff/leaprc.ff99SBildn,leaprc.gaff2"
  ions_parameters       = 1
  PBRadii               = 3
  temperature           = 298.15
  qh_entropy            = 0
  interaction_entropy   = 0
  c2_entropy            = 0
  assign_chainID        = 0
  exp_ki                = 0.0
  full_traj             = 0
  keep_files            = 0
  netcdf                = 0
  solvated_trajectory   = 0
  verbose               = 1
/

# 3D-RISM namelist variables
&rism
  closure               = "kh"
  gfcorrection          = 0
  pcpluscorrection      = 0
  noasympcorr           = 1
  buffer                = 14.0
  solvcut               = -1.0
  grdspc                = 0.5,0.5,0.5
  tolerance             = 1e-05
  ljTolerance           = -1.0
```

```
  asympKSpaceTolerance = -1.0
  treeDCF              = 1
  treeTCF              = 1
  treeCoulomb          = 0
  treeDCFMAC           = 0.1
  treeTCFMAC           = 0.1
  treeDCFOrder         = 2
  treeTCFOrder         = 2
  treeDCFN0            = 500
  treeTCFN0            = 500
  mdiis_del            = 0.7
  mdiis_nvec           = 5
  mdiis_restart        = 10.0
  maxstep              = 10000
  npropagate           = 5
  polardecomp          = 0
  entropicdecomp       = 0
  rism_verbose         = 0
/
```

2 Structure-Molecular Dynamics Interface Statistics

Table S1: Detailed numbers of protein-ligand complexes from the PDBbind 2020 refined set that were successfully converted or rejected by the structure-molecular dynamics interface.

Description	Number
**Complexes in the PDBbind 2020 refined set**	**5316**
**Successfully converted**	**4491**
**Rejected for scientific reasons**	**613**
**Protein beyond the interface's scope**	**451**
Membrane protein	81
Non-standard force field parameters required	370
Non-standard amino acids	*178*
Unsupported complexated ions	*191*
Unsupported co-solvents	*1*
**Non-repairable flaws in protein input**	**63**
Missing atoms close to/at the ligand binding site	50
Inconsistent sequence information in PDB file	5
Partially unknown sequence in official FASTA file	8
**Ligand topology could not be generated**	**99**
STaGE invocation ended in fatal error	74
Peptide ligand with missing atoms	25
**Technical errors caught by built-in checks**	**212**
**CONECT statements and disulphide bonds**	**35**
CONECT statements analysed incorrectly	1
Number of disulphide bonds not preserved	34
**Protein structure repair unsuccessful**	**107**
Alignment errors	15
Errors during the detection of structural gaps	49
Modelling with ProMod 3.3	43
Stereo-chemical problems	*17*
Large RMSD to experimental structure	*16*
Still gaps after modelling	*10*
**Invocation of GROMACS routines**	**64**
grompp	57
mdrun	7
**Other sources (error 404, ...)**	**6**

As a concluding remark on the BAP workflow performances, we mention that since PDBbind is a dataset of protein-ligand complexes that could be crystallised and thus have a negative binding affinity, complexes returned having ABFE>0 estimates are false negatives. Protein-ligand complexes with positive ABFEs should have dissociated in the molecular dynamics

simulations, which indicates that the potential used in the molecular dynamics simulations and in the implicit-solvent calculations may be inconsistent for these complexes. For these reasons, the complexes were also excluded from the data used for training the machine-learning model, as reported in Section 4.6.

On the other side, if dissociation actually occurs at the MD stage, the ABFE calculated with the implicit-solvation method amounts to zero under the Single-Trajectory Protocol (STP) and the neglected Entropy approximations, since the separate solvation free energies for the protein and the ligand become identical to the solvation free energy of the complex if the protein and the ligand are far apart. Therefore, on top of complexes returned having ABFE>0 values, we have removed from R_p, R_s evaluation also complexes returned having ABFE=0 (computation artefacts), as declared in Section 4.5.

Nonetheless, it is worth noting that complexes returned having ABFE=0 and complexes returned having ABFE>0 values might be treated differently for the following reason:

- ABFE=0 means that the complex dissociated during MD, we had no bound form to analyse in the implicit-solvent calculation such that it was technically impossible to provide an ABFE estimate. In that sense, these complexes failed a workflow stage (MD in this case) like the complexes that were rejected by the structure-MD interface.

- In contrast, ABFE>0 means that the implicit-solvent calculation was technically possible (there still was a bound form to evaluate with IS methods after MD). Although the predicted ABFE may be unreliable, these complexes technically passed all workflow stages. For this reason, they are inaccurate and unreliable but still technically valid results, and may be used to assess workflow performance.

Finally, we report that BAP workflow performance on complexes returned having $ABFEs \leq 0$ is $R_p = 0,368$ (95% CI [0.337, 0.398]).

3 Distribution of Binding Affinities in the PDBbind 2020 Refined Set and in Our Validation Set

Table S2: Distribution of K_d and K_i values across affinity ranges in the datasets. 'Refined' refers to the PDBbind v2020 refined set, while "Validation" refers to the independent validation set used in this work to validate the implicit-solvent calculations of the Binding Affinity Prediction (BAP) and Pose Selector (PS) workflows.

**Affinity Range**	**Refined**(K_D)	**Validation**(K_D)	**Refined**(K_i)	**Validation**(K_i)
1–10 pM	11	0	49	0
10–100 pM	31	0	94	0
100 pM–1 nM	95	1	199	1
1–10 nM	197	7	413	2
10–100 nM	472	12	487	5
100 nM–1 µM	579	10	425	2
1–10 µM	567	8	327	1
10–100 µM	457	6	286	3
100 µM–1 mM	255	1	173	2
1–10 mM	119	2	80	0

4 Absolute Binding Free Energy Estimates Computed during Workflow Validation

Table S3: Comparison of absolute binding free energy (ABFE) estimates provided by different implicit-solvation models for the protein-ligand complexes in the validation set for the PS workflow. Here the solute and solvent dielectric constants for the generalised Born surface area (GBSA) and non-linear Poisson-Boltzmann (NLPB) models are 3 & 80, respectively. This data represent the data points shown in Figure 4 of the main manuscript.

**PDB ID**	$\Delta G_{\mathbf{exp}}$	**Calculated ΔG Values**		
		**First Frame Analysis**		
	kJ/mol	GBSA	3D-RISM	NLPB
1ceb	-34.46	-23.61	-23.15	-15.40
2i3i	-41.93	-41.13	-31.66	-21.94
2vxn	-28.72	-17.42	-14.09	-9.33
2xnb	-39.23	-37.92	-7.780	-6.67
3eqr	-49.97	-51.39	-20.09	-17.00
3f17	-49.57	-27.13	-5.624	-1.87
3f18	-42.50	-14.62	-4.145	2.13
3f7h	-40.72	-43.90	-28.47	-22.40
3f7i	-42.16	-41.76	-30.21	-21.33
3hl5	-25.67	-33.23	-18.11	-14.78
3ikd	-38.77	-28.84	-13.09	-9.45
3kiv	-26.99	-18.13	-11.64	-8.94
3mho	-37.27	-19.83	-6.833	2.65
3nkk	-26.99	-20.85	-12.29	-11.00

Continued on next page

Table S3 – continued from previous page

PDB ID	$\Delta G_{\mathbf{exp}}$	Calculated ΔG Values First Frame Analysis		
	kJ/mol	GBSA	3D-RISM	NLPB
3pyy	-39.40	-31.02	-15.18	-14.43
3qgy	-44.80	-28.46	-6.789	-2.61
3s0d	-40.49	-21.65	-6.811	-4.95
3uw5	-45.09	-41.49	-28.63	-20.50
4ayq	-42.10	-16.67	-15.18	26.26
4css	-48.59	-24.74	-22.97	-9.79
4cst	-51.06	-29.33	-27.86	-13.63
4erf	-53.99	-25.92	-5.755	-4.45
4gj2	-49.39	-33.81	-18.38	-15.37
4jfk	-36.99	-34.19	-12.66	-7.64
4jfm	-31.47	-22.29	-0.043	-0.33
4kqp	-34.75	-30.24	-32.99	-16.78
4pmm	-43.48	-52.40	-18.70	-23.14
4q08	-36.18	-27.55	-13.31	11.25
4q6e	-35.49	-19.21	-2.27	1.08
4tkb	-32.10	-26.22	-15.58	-12.29
4tkj	-34.69	-35.37	-18.08	-15.48
4wn5	-42.56	-41.84	-13.94	-16.94
4x5p	-47.15	-28.97	-28.48	-13.63
4x5q	-51.69	-28.10	-24.44	-12.56

Continued on next page

Table S3 – continued from previous page

PDB ID	$\Delta G_{\mathbf{exp}}$	Calculated ΔG Values First Frame Analysis		
	kJ/mol	GBSA	3D-RISM	NLPB
5hbs	-44.40	-38.69	-13.64	-11.78
5k8s	-47.21	-36.17	-21.61	-16.21
5meh	-36.30	-18.56	-25.70	-10.49
5ne5	-42.56	-16.59	-17.63	21.43
5nk3	-42.62	-38.08	-7.61	-12.36
5nkg	-44.11	-41.25	-9.39	-11.81
5od1	-34.23	-29.92	-17.08	-10.30
5oh3	-22.22	-22.00	-19.87	-13.20
5oh4	-26.13	-17.57	-12.75	-9.54
5oh7	-25.10	-15.35	-9.63	-8.31
5oh9	-22.17	-17.44	-13.79	-10.89
5oha	-28.08	-15.54	-11.40	-8.79
5vd2	-40.78	-33.81	-7.380	-6.05
5wa9	-38.83	-36.56	-28.52	-12.26
5wxh	-35.32	-41.76	-9.09	-9.92
5yj8	-22.86	-0.152	0.35	0.072
6b8y	-55.48	-37.31	-17.55	-15.05
6ce6	-29.29	-21.01	-5.04	-6.93
6ced	-33.43	-23.97	-14.70	-13.79
6fgg	-27.39	-20.50	-10.37	-7.77

Continued on next page

Table S3 – continued from previous page

**PDB ID**	$\Delta G_{\mathbf{exp}}$	**Calculated ΔG Values**		
		**First Frame Analysis**		
	kJ/mol	GBSA	3D-RISM	NLPB
6fhu	-15.16	-19.64	-8.35	-6.43
6fmc	-33.77	-17.49	-14.36	-6.67
6mj7	-16.19	-17.09	-16.71	-10.03
6n3x	-29.23	-31.98	-14.03	-9.03
6nfy	-44.80	-36.43	-8.87	-11.33
6np4	-25.50	-46.94	-17.96	-12.61
6qau	-42.96	-46.97	-20.48	-16.00
6qls	-30.73	-28.07	-17.01	-10.26
6sdc	-51.12	-60.05	-24.38	-20.95

Table S4: Comparison of ABFE estimates provided by different implicit-solvation models for the protein-ligand complexes in the validation set for the BAP workflow. Here the solute and solvent dielectric constants for GBSA and NLPB are 3 & 80, respectively. This data represent the data points shown in Figure 5 of the main manuscript.

**PDB ID**	$\Delta G_{\mathbf{exp}}$	**Calculated ΔG Values**		
	kJ/mol	GBSA	3D-RISM	NLPB
1ceb	-34.46	-20.53	-17.34	-10.80
2i3i	-41.93	-40.44	-24.83	-18.95
2vxn	-28.72	-2.20	-7.92	-2.19
2xnb	-39.23	-34.35	-7.89	-6.92
3eqr	-49.97	-51.24	-16.37	-15.85
3f17	-49.57	-29.31	-6.81	-7.13
3f18	-42.51	-10.98	-6.21	-2.62
3f7h	-40.73	-40.87	-24.94	-18.75
3f7i	-42.16	-41.41	-26.00	-19.91
3hl5	-25.68	-30.96	-19.51	-13.96
3ikd	-38.77	-29.88	-18.80	-13.76
3kiv	-27.00	-8.40	-5.70	-5.52
3mho	-37.28	-25.62	-8.65	-0.98
3nkk	-27.00	-18.08	-10.30	-7.77
3pyy	-39.40	-29.84	-8.92	-10.05
3qgy	-44.80	-25.34	-3.35	-5.16
3s0d	-40.50	-28.78	-13.57	-11.83
3uw5	-45.09	-39.94	-24.31	-18.30

Continued on next page

Table S4 – continued from previous page

PDB ID	$\Delta G_{\mathbf{exp}}$	Calculated ΔG Values		
	kJ/mol	GBSA	3D-RISM	NLPB
4ayq	-42.10	-8.58	-8.42	23.40
4css	-48.60	-26.29	-18.80	-8.08
4cst	-51.07	-24.55	-20.15	-8.59
4erf	-53.99	-30.43	-6.21	-9.96
4gj2	-49.40	-32.45	-11.90	-10.13
4jfk	-36.99	-24.22	-8.17	-6.81
4jfm	-31.48	-28.33	-7.93	-6.21
4kqp	-34.75	-24.51	-23.79	-10.51
4pmm	-43.48	-50.28	-15.72	-21.68
4q08	-36.19	-27.74	-7.44	1.02
4q6e	-35.50	-9.92	-3.69	1.98
4tkb	-32.11	-22.98	-10.37	-7.72
4tkj	-34.69	-28.63	-12.44	-9.84
4wn5	-42.56	-40.11	-9.87	-16.78
4x5p	-47.16	-23.57	-20.98	-8.67
4x5q	-51.70	-23.05	-19.20	-8.43
5hbs	-44.40	-35.24	-6.43	-6.90
5k8s	-47.22	-36.50	-22.20	-16.50
5meh	-36.30	-18.54	-22.00	0.04
5ne5	-42.56	-12.59	-11.42	16.67
5nk3	-42.62	-39.90	-12.57	-13.77

Continued on next page

Table S4 – continued from previous page

PDB ID	$\Delta G_{\mathbf{exp}}$	Calculated ΔG Values		
	kJ/mol	GBSA	3D-RISM	NLPB
5nkg	-44.12	-34.14	-14.43	-13.72
5od1	-34.24	-13.32	-6.71	-4.07
5oh3	-22.23	-19.78	-12.55	-10.20
5oh4	-26.14	-17.09	-11.88	-9.21
5oh7	-25.10	-15.24	-11.75	-8.68
5oh9	-22.17	-17.05	-13.24	-10.24
5oha	-28.09	-15.81	-11.94	-8.69
5vd2	-40.78	-32.45	-8.79	-7.29
5wa9	-38.83	-10.94	-13.91	-5.02
5wxh	-35.33	-48.78	-26.50	-20.99
5yj8	-22.86	-0.17	-0.15	0.18
6b8y	-55.49	-36.90	-14.03	-12.98
6ce6	-29.30	-13.31	-5.75	-5.17
6ced	-33.43	-19.97	-12.27	-8.86
6fgg	-27.40	-20.04	-7.94	-5.43
6fhu	-15.16	-2.05	-3.90	-2.19
6fmc	-33.78	-20.11	-9.37	-4.23
6mj7	-16.20	-6.73	-10.98	-4.19
6n3x	-29.24	-28.70	-17.86	-8.04
6nfy	-44.80	-33.30	-11.85	-8.38
6np4	-25.50	-43.68	-40.83	-25.13

Continued on next page

Table S4 – continued from previous page

**PDB ID**	$\Delta G_{\mathbf{exp}}$	**Calculated ΔG Values**		
	kJ/mol	GBSA	3D-RISM	NLPB
6qau	-42.97	-47.74	-17.76	-15.42
6qls	-30.73	-26.29	-18.65	-8.61
6sdc	-51.12	-54.67	-19.48	-18.13

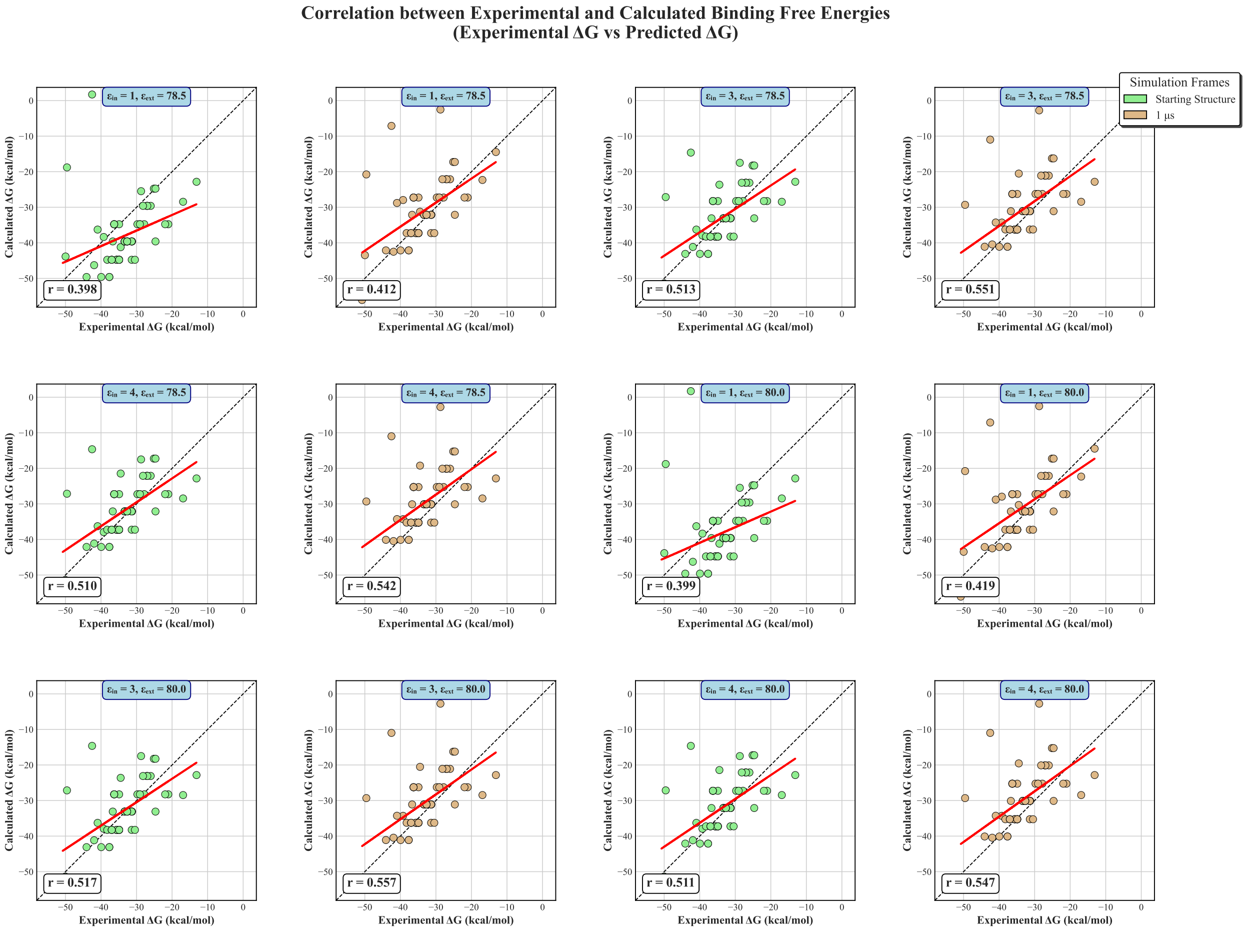

Figure S1: Correlation analysis between experimental and calculated ΔG values using the generalized Born model. Scatter plots show the relationship between experimental, pK_a-derived binding free energies and GB model predictions across 12 different parameter combinations. Each subplot represents a specific combination of solute dielectric constant ($\varepsilon_{\text{in}} = 1$, 3, or 4) and solvent dielectric constant ($\varepsilon_{\text{ext}} = 78.5$ or 80.0) for both starting structure only (green) and 1 μs-long molecular dynamics simulations (tan). Pearson correlation coefficients (r) are displayed for each parameter set. Red lines indicate linear regression fits. The highest correlation ($r = 0.557$) was achieved with $\varepsilon_{\text{in}} = 3$, $\varepsilon_{\text{ext}} = 80.0$ using 1 μs-long simulations, demonstrating optimal GB model performance under these conditions.

Table S5: ABFE estimates provided by GBSA with solute and solvent dielectric constants set to 3 & 80, respectively, as a function of the simulated time per trajectory to assess the impact of configurational sampling in the MD simulation on the accuracy of the ABFE estimate. This data explains the Pearson correlation coefficients listed in Table 1 of the main manuscript.

**PDB ID**	$\Delta G_{\mathbf{exp}}$	**Calculated ΔG Values**				
	kJ/mol	1st Frame	250 ns	500 ns	750 ns	1000 ns
1ceb	-34.46	-23.62	-20.95	-20.93	-20.85	-20.53
2i3i	-41.93	-41.13	-40.51	-40.46	-40.47	-40.44
2vxn	-28.72	-17.43	-4.38	-3.68	-4.27	-2.20
2xnb	-39.23	-37.92	-34.47	-34.69	-34.51	-34.35
3eqr	-49.97	-51.39	-52.34	-52.20	-51.72	-51.24
3f17	-49.57	-27.14	-29.62	-29.51	-29.32	-29.31
3f18	-42.51	-14.62	-17.89	-14.58	-12.83	-10.98
3f7h	-40.73	-43.91	-40.93	-40.69	-40.78	-40.87
3f7i	-42.16	-41.77	-40.95	-41.16	-41.18	-41.41
3hl5	-25.68	-33.24	-29.27	-30.07	-30.69	-30.96
3ikd	-38.77	-28.84	-28.58	-29.16	-29.71	-29.88
3kiv	-27.00	-18.13	-17.10	-14.96	-12.11	-8.40
3mho	-37.28	-19.83	-22.51	-22.91	-23.99	-25.62
3nkk	-27.00	-20.86	-18.25	-18.15	-18.09	-18.08
3pyy	-39.40	-31.03	-30.02	-29.85	-29.82	-29.84
3qgy	-44.80	-28.46	-30.36	-28.31	-26.21	-25.34
3s0d	-40.50	-21.66	-27.40	-28.32	-28.54	-28.78
3uw5	-45.09	-41.49	-39.72	-39.74	-39.79	-39.94

Continued on next page

Table S5 – continued from previous page

PDB ID	$\Delta G_{\mathbf{exp}}$	Calculated ΔG Values				
	kJ/mol	1st Frame	250 ns	500 ns	750 ns	1000 ns
4ayq	-42.10	-16.68	-10.57	-8.83	-8.75	-8.58
4css	-48.60	-24.75	-22.67	-22.52	-22.56	-26.29
4cst	-51.07	-29.33	-24.44	-24.72	-24.64	-24.55
4erf	-53.99	-25.93	-30.72	-30.23	-30.33	-30.43
4gj2	-49.40	-33.82	-32.56	-32.23	-32.40	-32.45
4jfk	-36.99	-34.19	-30.08	-30.30	-28.53	-24.22
4jfm	-31.48	-22.29	-29.10	-29.33	-29.32	-28.33
4kqp	-34.75	-30.25	-24.38	-24.44	-24.39	-24.51
4pmm	-43.48	-52.40	-49.66	-49.92	-49.85	-50.28
4q08	-36.19	-27.55	-23.89	-26.09	-27.06	-27.74
4q6e	-35.50	-19.22	-13.91	-11.29	-10.46	-9.92
4tkb	-32.11	-26.23	-22.86	-22.95	-22.92	-22.98
4tkj	-34.69	-35.37	-28.28	-29.17	-29.29	-28.63
4wn5	-42.56	-41.85	-37.89	-39.25	-39.70	-40.11
4x5p	-47.16	-28.97	-22.70	-23.47	-23.55	-23.57
4x5q	-51.70	-28.11	-23.75	-23.38	-23.04	-23.05
5hbs	-44.40	-38.70	-33.90	-34.86	-35.07	-35.24
5k8s	-47.22	-36.17	-37.27	-36.57	-36.52	-36.50
5meh	-36.30	-18.56	-18.62	-18.32	-18.41	-18.54
5ne5	-42.56	-16.60	-14.11	-13.68	-13.48	-12.59
5nk3	-42.62	-38.09	-39.98	-40.03	-40.12	-39.90

Continued on next page

Table S5 – continued from previous page

PDB ID	$\Delta G_{\mathbf{exp}}$	Calculated ΔG Values				
	kJ/mol	1st Frame	250 ns	500 ns	750 ns	1000 ns
5nkg	-44.12	-41.26	-32.65	-34.79	-34.37	-34.14
5od1	-34.24	-29.93	-22.99	-18.15	-15.57	-13.32
5oh3	-22.23	-22.01	-19.78	-19.77	-19.77	-19.78
5oh4	-26.14	-17.58	-17.05	-17.08	-17.08	-17.09
5oh7	-25.10	-15.35	-15.27	-15.19	-15.22	-15.24
5oh9	-22.17	-17.45	-17.15	-17.07	-17.05	-17.05
5oha	-28.09	-15.55	-15.94	-15.86	-15.86	-15.81
5vd2	-40.78	-33.81	-33.58	-33.13	-32.86	-32.45
5wa9	-38.83	-36.57	-22.56	-15.80	-12.53	-10.94
5wxh	-35.33	-41.77	-49.53	-49.35	-49.05	-48.78
5yj8	-22.86	-0.15	-0.17	-0.17	-0.17	-0.17
6b8y	-55.49	-37.32	-37.08	-37.04	-37.05	-36.90
6ce6	-29.30	-21.01	-22.02	-18.61	-14.97	-13.31
6ced	-33.43	-23.98	-21.39	-21.10	-21.29	-19.97
6fgg	-27.40	-20.51	-23.26	-21.09	-20.28	-20.04
6fhu	-15.16	-19.64	-6.48	-4.04	-2.71	-2.05
6fmc	-33.78	-17.49	-20.70	-21.56	-21.39	-20.11
6mj7	-16.20	-17.09	-9.64	-9.30	-7.74	-6.73
6n3x	-29.24	-31.98	-29.10	-29.14	-28.76	-28.70
6nfy	-44.80	-36.43	-33.53	-33.46	-33.21	-33.30
6np4	-25.50	-46.95	-42.65	-43.15	-43.74	-43.68

Continued on next page

Table S5 – continued from previous page

PDB ID	$\Delta G_{\mathbf{exp}}$	Calculated ΔG Values				
	kJ/mol	1st Frame	250 ns	500 ns	750 ns	1000 ns
6qau	-42.97	-46.98	-47.36	-47.58	-47.46	-47.74
6qls	-30.73	-28.07	-26.17	-26.26	-26.25	-26.29
6sdc	-51.12	-60.05	-54.88	-55.44	-55.06	-54.67

As a concluding remark, we report that while a few complexes (e.g., 2vxn, 3f18, 3kiv, 3qgy, and 6np4) deviate further from the initial ΔG estimate as sampling increases, most systems display relatively stable ΔG estimates with increasing trajectory length. A detailed inspection of the corresponding trajectories reveals that in these outlier cases the ligand progressively departs from the initial binding mode, losing key protein–ligand interactions and sampling alternative, non-native conformations. Since this behaviour is confined to a small subset of systems while the majority stays in its bound form, the resulting changes in Pearson correlation are small (≈ 0.04) and can be attributed to specific outliers rather than to a systematic dependence on sampling time. This behaviour is consistent with the known limitations of endpoint methods such as MM/GBSA, where inaccuracies may arise from force-field limitations, pose drift, or imperfect treatment of entropic and solvent effects.

References

(1) Wang, R.; Fang, X.; Lu, Y.; Yang, C.-Y.; Wang, S. The PDBbind database: methodologies and updates. *J. Med. Chem.* **2005**, *48*, 4111–4119.

(2) Madhavi Sastry, G.; Adzhigirey, M.; Day, T.; Annabhimoju, R.; Sherman, W. Protein and ligand preparation: parameters, protocols, and influence on virtual screening enrichments. *J. Comput.-Aided Mol. Des.* **2013**, *27*, 221–234.

(3) Eastman, P.; Swails, J.; Chodera, J. D.; McGibbon, R. T.; Zhao, Y.; Beauchamp, K. A.; Wang, L.-P.; Simmonett, A. C.; Harrigan, M. P.; Stern, C. D.; others OpenMM 7: Rapid development of high performance algorithms for molecular dynamics. *PLOS Comput. Biol.* **2017**, *13*, e1005659.

(4) Schrödinger Schrödinger Release 2023-4: LigPrep, Schrödinger, LLC, New York, NY,.

(5) Gadioli, D.; Vitali, E.; Ficarelli, F.; Latini, C.; Manelfi, C.; Talarico, C.; Silvano, C.; Cavazzoni, C.; Palermo, G.; Beccari, A. R. EXSCALATE: An Extreme-Scale Virtual Screening Platform for Drug Discovery Targeting Polypharmacology to Fight SARS-CoV-2. *IEEE Trans. Emerg. Top. Comput.* **2023**, *11*, 170–181.

(6) Bernhofer, M.; Rost, B. TMbed: transmembrane proteins predicted through language model embeddings. *BMC Bioinform.* **2022**, *23*, 326.

(7) Rodrigues, J. P. G. L. M.; Teixeira, J. M. C.; Trellet, M.; Bonvin, A. M. J. J. pdb-tools: a swiss army knife for molecular structures. *F1000Research* **2018**, *7*, 1961.

(8) Cock, P. J. A.; Antao, T.; Chang, J. T.; Chapman, B. A.; Cox, C. J.; Dalke, A.; Friedberg, I.; Hamelryck, T.; Kauff, F.; Wilczynski, B.; de Hoon, M. J. L. Biopython: freely available Python tools for computational molecular biology and bioinformatics. *Bioinformatics* **2009**, *25*, 1422–1423.

(9) Biasini, M.; Mariani, V.; Haas, J.; Scheuber, S.; Schenk, A. D.; T., S.; Philippsen, A. OpenStructure: A flexible software framework for computational structural biology. *Bioinformatics* **2010**, *26*, 2626–2628.

(10) Biasini, M.; Schmidt, T.; Bienert, S.; Mariani, V.; Studer, G.; Haas, J.; Johner, N.; Schenk, A. D.; Philippsen, A.; T., S. OpenStructure: an integrated software framework for computational structural biology. *Acta Cryst.* **2013**, *69*, 701–709.

(11) Kabsch, W. A solution for the best rotation to relate two sets of vectors. *Acta Cryst.* **1976**, *32*, 922–923.

(12) Crouse, D. F. On implementing 2D rectangular assignment algorithms. *IEEE Trans. Aeros. Electron. Syst.* **2016**, *52*, 1679–1696.

(13) Abraham, M. J.; Murtola, T.; Schulz, R.; Páll, S.; Smith, J. C.; Hess, B.; Lindahl, E. GROMACS: High performance molecular simulations through multi-level parallelism from laptops to supercomputers. *SoftwareX* **2015**, *1–2*, 19–25.

(14) Páll, S.; Abraham, M. J.; Kutzner, C.; Hess, B.; Lindahl, E. In *Solving Software Challenges for Exascale*; Markidis, S., Laure, E., Eds.; 2015; Vol. 8759; pp 3–27.

(15) Pronk, S.; Páll, S.; Schulz, R.; Larsson, P.; Bjelkmar, P.; Apostolov, R.; Shirts, M. R.; Smith, J. C.; Kasson, P. M.; van der Spoel, D.; Hess, B.; Lindahl, E. GROMACS 4.5: a high-throughput and highly parallel open source molecular simulation toolkit. *Bioinformatics* **2013**, *29*, 845–854.

(16) Hess, B.; Kutzner, C.; van der Spoel, D.; Lindahl, E. GROMACS 4: Algorithms for Highly Efficient, Load-Balanced, and Scalable Molecular Simulation. *J. Chem. Theory Comput.* **2008**, *4*, 435–447.

(17) van der Spoel, D.; Lindahl, E.; Hess, B.; Groenhof, G.; Mark, A. E.; Berendsen, H. J. C. GROMACS: Fast, Flexible, and Free. *J. Comput. Chem.* **2005**, *26*, 1701–1718.

(18) Lindahl, E.; Hess, B.; van der Spoel, D. GROMACS 3.0: a package for molecular simulation and trajectory analysis. *J. Mol. Model.* **2001**, *7*, 306–317.

(19) Berendsen, H. J. C.; van der Spoel, D.; van Drunen, R. GROMACS: A message-passing parallel molecular dynamics implementation. *Comp. Phys. Comm.* **1995**, *91*, 43–56.

(20) Cornell, W. D.; Cieplak, P.; Bayly, C. I.; Gould, I. R.; Merz, K. M.; Jr.; Ferguson, D. M.; Spellmeyer, D. C.; Fox, T.; Caldwell, J. W.; Kollman, P. A. A Second Generation Force Field for the Simulation of Proteins, Nucleic Acids, and Organic Molecules. *J. Am. Chem. Soc.* **1995**, *117*, 5179–5197.

(21) Hornak, V.; Abel, R.; Okur, A.; Strockbine, B.; Roitberg, A.; Simmerling, C. Comparison of Multiple Amber Force Fields and Development of Improved Protein Backbone Parameters. *Proteins: Struct., Funct., Bioinf.* **2006**, *65*, 712–725.

(22) Lindorff-Larsen, K.; Piana, S.; Palmo, K.; Maragakis, P.; Klepeis, J. L.; Dror, R. O.; Shaw, D. E. Improved side-chain torsion potentials for the Amber ff99SB protein force field. *Proteins: Struct., Funct., Bioinf.* **2010**, *78*, 1950–1958.

(23) Jorgensen, W. L.; Chandrasekhar, J.; Madura, J. D.; Impey, R. W.; Klein, M. L. Comparison of simple potential functions for simulating liquid water. *J. Chem. Phys.* **1983**, *79*, 926–935.

(24) Wang, J.; Wolf, R. M.; Caldwell, J. W.; Kollman, P. A.; Case, D. A. Development and testing of a general amber force field. *J. Comput. Chem.* **2004**, *25*, 1157–1174.

(25) Batista, P. R.; Wilter, A.; Durham, E. H. A. B.; Pascutti, P. G. Molecular Dynamics Simulations Applied to the Study of Subtypes of HIV-1 Protease. *Cell Biochem. Biophys.* **2006**, *44*, 395–404.

(26) Sousa da Silva, A. W.; Vranken, W. F. ACPYPE - AnteChamber PYthon Parser interfacE. *BMC Res. Notes* **2012**, *5*, 367.

(27) Wang, J.; Wang, W.; Kollman, P. A.; Case, D. A. Automatic atom type and bond type perception in molecular mechanical calculations. *J. Mol. Graph.* **2006**, *25*, 247–260.

(28) Darden, T.; York, D.; Pedersen, L. Particle mesh Ewald: An N · log(N) method for Ewald sums in large systems. *J. Chem. Phys.* **1993**, *98*, 10089–10092.

(29) Bussi, G.; Donadio, D.; Parrinello, M. Canonical sampling through velocity rescaling. *J. Chem. Phys.* **2007**, *126*, 014101.

(30) Bernetti, M.; Bussi, G. Pressure control using stochastic cell rescaling. *J. Chem. Phys.* **2020**, *153*, 114107.

(31) Hess, B.; Bekker, H.; Berendsen, H. J. C.; Fraaije, J. G. E. M. LINCS: A linear constraint solver for molecular simulations. *J. Comp. Chem.* **1997**, *18*, 1463–1472.

(32) Hess, B. P-LINCS: A parallel linear constraint solver for molecular simulation. *J. Chem. Theory Comput.* **2007**, *4*, 116–122.

(33) Onufriev, A.; Bashford, D.; Case, D. A. Exploring protein native states and large-scale conformational changes with a modified generalized Born model. *Proteins: Struct., Funct., Bioinf.* **2004**, *55*, 383–394.

(34) Lu, Q.; Luo, R. A Poisson–Boltzmann dynamics method with nonperiodic boundary condition. *J. Chem. Phys.* **2003**, *119*, 11035–11047.

(35) Luo, R.; David, L.; Gilson, M. Accelerated Poisson-Boltzmann calculations for static and dynamic systems. *J. Comput. Chem.* **2002**, *23*, 1244–53.

(36) Wang, J.; Cai, Q.; Xiang, Y.; Luo, R. Reducing Grid Dependence in Finite-Difference Poisson-Boltzmann Calculations. *J. Chem. Theory Comput.* **2012**, *8*, 2741–2751.

(37) Davis, M. E.; McCammon, J. A. Electrostatics in biomolecular structure and dynamics. *Chem. Rev.* **1990**, *90*, 509–521.

(38) Floris, F.; Tomasi, J. Evaluation of the dispersion contribution to the solvation energy. A simple computational model in the continuum approximation. *J. Comput. Chem.* **1989**, *10*, 616–627.

(39) Tan, C.; Tan, Y.-H.; Luo, R. Implicit Nonpolar Solvent Models. *J. Phys. Chem. B* **2007**, *111*, 12263–12274.
